# A Generalized Information-Theoretic Framework for the Emergence of Hierarchical Abstractions in Resource-Limited Systems

**DOI:** 10.3390/e24060809

**Published:** 2022-06-09

**Authors:** Daniel T. Larsson, Dipankar Maity, Panagiotis Tsiotras

**Affiliations:** 1 D. Guggenheim School of Aerospace Engineering, Georgia Institute of Technology, Atlanta, GA 30332-0150, USA; 2 Department of Electrical and Computer Engineering, The University of North Carolina at Charlotte, Charlotte, NC 28223-0001, USA; dmaity@uncc.edu; 3 D. Guggenheim School of Aerospace Engineering, Institute for Robotics and Intelligent Machines, Georgia Institute of Technology, Atlanta, GA 30332-0150, USA; tsiotras@gatech.edu

**Keywords:** hierarchical abstractions, hierarchical tree abstractions, information bottleneck method, information-theoretic privacy, multi-resolution abstractions

## Abstract

In this paper, a generalized information-theoretic framework for the emergence of multi-resolution hierarchical tree abstractions is developed. By leveraging ideas from information-theoretic signal encoding with side information, this paper develops a tree search problem which considers the generation of multi-resolution tree abstractions when there are multiple sources of relevant and irrelevant, or possibly confidential, information. We rigorously formulate an information-theoretic driven tree abstraction problem and discuss its connections with information-theoretic privacy and resource-limited systems. The problem structure is investigated and a novel algorithm, called G-tree search, is proposed. The proposed algorithm is analyzed and a number of theoretical results are established, including the optimally of the G-tree search algorithm. To demonstrate the utility of the proposed framework, we apply our method to a real-world example and provide a discussion of the results from the viewpoint of designing hierarchical abstractions for autonomous systems.

## 1. Introduction

Driven by the human ability to discern pertinent details from immense amounts of perceptual information, the process of identifying task-relevant structures from data has long been considered a cornerstone to the development of intelligent systems [1,2,3,4]. To this end, researchers in the autonomous systems community have spend a great deal of effort studying abstractions, which is a problem generally viewed as an information-removal procedure to discard details that are not relevant for a given task [1,2,3,4,5]. The central motivation for the employment of abstractions is to simplify the problem domain by removing details that can be safely ignored, thereby creating a new representation of the problem for which reasoning and decision making requires fewer computational resources [1,2,3]. Despite their importance, autonomous systems thus far seldom design abstractions on their own, instead relying on system designers and prior domain knowledge to provide hand-crafted rules for the emergence of abstractions as a function of task in various domains [3,5]. In spite of these shortcomings, abstractions have seen wide-spread use in a number of autonomous systems applications.

Perhaps the most notable field of research where abstractions have seen particular success is within the planning community [1]. Examples of work that employ the power of abstract representations in planning for autonomous systems include [6,7,8,9,10,11,12,13,14]. The idea of utilizing abstractions in planning is to form reduced graphs on which classical search algorithms, such as A${}^{*}$ and Dijkstra, are implemented. By reducing the number of vertices in the graph, the computational burden of executing these search algorithms is reduced. However, while the cited works all leverage graph abstractions to ease the computational cost of planning, the methods by which they generate these abstractions differ. For example, in [7,8,9,10,11] the environment abstractions are created via the wavelet transform. In contrast, the works of [12,13,14] generate abstractions of the environment in the form of multi-resolution quadtree and octree data structures. Notably, the work of [12,13] develops a framework that incorporates sensor uncertainty in robotic systems by merging ideas from multi-resolution planning and probabilistic tree structures introduced in [15]. Today, the use of probabilistic trees in robotics is ubiquitous, and has led to the development of open-source software packages for their implementation [16].

Motivated by the possibly dynamic nature of the environment as well as sensing limitations inherent to autonomous systems, the abstractions employed in all the aforementioned works maintain high resolution nearest the autonomous agent (e.g., robotic ground vehicle), while aggregating other portions of the environment at various resolution levels. In this way, the region nearest to the vehicle is considered the most relevant, and thus preserved through the process of abstraction. To strike a balance between path-optimality (system performance) and the computational cost of planning, agents recursively re-plan as they traverse the world.

The design of abstractions has also been considered by information theorists in the context of optimal signal encoding for communication over capacity-limited channels [17]. In order to formulate mathematical optimization problems that yield optimal encoders it is required to identify the relevant structure of the original signal necessary to guarantee that a satisfactory system performance can be achieved. To this end, the framework of rate-distortion theory approaches the optimal encoder problem by measuring the degree of compression via the mutual information between the compressed representation and the original signal, whereas the performance of the system is quantified by a user-provided distortion function [17]. In this way, the distortion function implicitly specifies which aspects of the original signal are relevant, and should be retained, in order to guarantee low distortion. A notable drawback to the rate-distortion framework is, however, the need to specify the distortion function, which may be difficult and non-intuitive for a given task [18].

In contrast, the information bottleneck (IB) method developed in [18] approaches the optimal encoder problem to preserve relevant information more directly. That is, the IB method considers an optimal encoder problem where the degree of achieved compression is captured by the mutual information between the compressed and original signals, and the model quality is measured by the mutual information between the compressed representation and an auxiliary variable which is assumed to contain task-relevant information. The IB approach is entirely data-driven, requiring only the joint distribution of the original signal and relevant information (i.e., the data) in order to be applied.

Owing to its general statistical formulation, the IB method, or some variation of it, has been considered in a number of studies [19,20,21,22,23,24,25,26]. Among these works, reference [19] develops an approach to obtain deterministic encoders to an IB-like problem motivated by reducing the number of clusters in the compressed space as opposed to designing encoders for communication. Consequently, the deterministic IB [19] measures the degree of achieved compression not by the mutual information between the original and compressed representations, as in communication systems, but by the entropy of the reduced space. The work of [20] considers the IB problem with side-information, allowing for both relevant and irrelevant structures to be provided to aid the identification of task-relevant information during the creation of signal encoders. The authors of [21,23] consider a multivariate extension of the IB principle, employing the use of Bayesian networks to specify the compression-relevance relations between the random variables to be maintained through the abstraction process. It should be noted that, while it does not directly employ the IB principle in its formulation, the empirical coordination problem [27,28] considers an information-theoretic compression problem over a graph, where interconnections between vertices represent communication links that agents may use to correlate their sequence of outcomes. Much like the multi-IB method [21,23], the network (communication) topology specifies the statistical dependencies that are possible in the empirical coordination problem. Observe, however, that the objective of the empirical coordination problem is to characterize the set of achievable joint distributions that are possible with various network topologies and communication (code) rates between vertices, whereas the multi-IB problem is a generalization of the encoder-design problem considered by the IB method to multivariate settings where the Bayesian networks are used to specify the relationships between source, reproduction and prediction (relevance) variables.

Other variants of the IB principle include the work in [22], where the authors consider the development of a bottom-up, agglomerative, hard clustering approach that employs the IB objective in determining which clusters to myopically merge at each step of the proposed algorithm. In related work inspired by the AIB problem, the authors of [29] exploit the structure of the AIB merging rule to design algorithms that form compressed representations of images by performing a sequence of greedy merges based on minimizing the stage-wise loss of relevant information at each iteration. Crucially, however, the algorithms developed in [29] do not consider the IB problem as they aim to design a sequence of myopic merges so as to minimize the loss of only relevant information, as compared with the much more challenging IB problem of simultaneously balancing information retention *and* information-theoretic compression. Moreover, in contrast to the work presented in this paper, the algorithms developed in [29] are not accompanied by theoretical performance guarantees that certify the optimality of the abstractions, nor are the methods readily extendable to cases where information from multiple sources must be considered in the design of compressed representations. Finally, the research conducted by the authors of [24] considers the IB problem in the setting of jointly-Gaussian data. More specifically, the authors of [24] established that when the original signal and the auxiliary (relevant) variable are jointly Gaussian, the solution to the IB problem is a noisy linear projection. For completeness, we note that when the data are not jointly Gaussian, or are described by a general probability density function, a solution to the IB problem is difficult to obtain. However, a number of studies have proposed methods leveraging variational inference in order to obtain approximate solutions to the IB problem in these cases [30,31,32,33].

Employing a unified viewpoint between abstractions in autonomy and those driven by information-theoretic principles, the authors of [34,35,36,37] developed frameworks for the emergence of abstractions in autonomous systems via methods inspired by information-theoretic signal compression. For example, the work of [34] employs the use of the IB principle to generate multi-resolution quadtree abstractions for planning, developing a framework that couples environment resolution, information and path value. Moreover, the research conducted in [35] utilizes environment abstractions to reduce the computational cost of evaluating mutual-information objective functions in active sensing applications. Of the reviewed works, those most closely related to the developments in this paper is that of [36,37], where the authors develop algorithms to select multi-resolution trees that are optimal with respect to the IB objective in both the soft-constrained (Lagrangian) [36] and hard-constrained [37] settings of the IB problem.

Inspired by the recent developments in information-theoretic driven approaches for generating abstractions for autonomous agents for the purposes of planning, the contribution of this paper is the development of a generalized information-theoretic framework that allows for multi-resolution tree abstractions to be obtained when multiple sources of relevance and irrelevance are specified. The incorporation of irrelevant information allows for connections between our framework and notions of information-theoretic privacy. Moreover, our generalized approach allows for abstractions to be refined by removing aspects of the relevant variables that are correlated with the irrelevant information structure, thus allowing for more compressed representations to emerge. This is especially critical in resource-constrained systems, which must make the best use of scarce on-board memory and bandwidth-limited communication channels.

The remainder of the paper is organized as follows. We begin in Section 3 with a brief overview of information-theoretic signal compression and detail the connection between hierarchical trees and signal encoders. Section 4 contains our formal problem statement. We propose and discuss solution approaches in Section 5. In Section 6, we present a discussion and comparison between the information-bottleneck method and the information-bottleneck problem with side-information (IBSI) in the setting of hierarchical tree abstractions. Examples and results are discussed in Section 7 before concluding remarks in Section 8. Proofs for the theoretical results presented in this paper are provided in the appendices.

## 2. Notation

Let R denote the set of real numbers and, for any integer n>0, let $R^{n}$ denote the *n*-dimensional Euclidean space. The set of non-negative real numbers is denoted by $R_{+}=\left\{x\in R:x\geq 0\right\}$. For any vector $x\in R^{n}$, ${\left[x\right]}_{i}$ is the ith element of the vector *x* for i∈{1,…,n}. For any integer n>0, the collection of all non-negative *n*-dimensional vectors is denoted $R_{+}^{n}=\left\{x\in R^{n}:{\left[x\right]}_{i}\geq 0,1\leq i\leq n\right\}$. Given any two vectors $x,y\in R^{n}$, the notation x≤y is understood component-wise; that is x≤y implies ${\left[x\right]}_{i}\leq {\left[y\right]}_{i}$ for all i∈{1,…,n}. Unless otherwise stated, all logarithms are base *e*.

## 3. Preliminaries

The development of a framework for generating information-theoretic multi-resolution abstractions requires the introduction of concepts from both information theory and graph theory in order to rigorously define trees and encoder problems. We begin by introducing necessary topics from information theory before proceeding to introduce hierarchical trees and their connection to multi-resolution representations of the environment. In the interest of succinctness, we only introduce the relevant topics from information theory necessary for the developments of our framework, and refer the interested readers to [17,38] for a more comprehensive exposition of information theoretic principles and classical signal compression frameworks. We close this section by elucidating how multi-resolution trees can be viewed as deterministic encoders having a special structure, thereby allowing us to employ information-theoretic concepts from signal encoding theory to formulate the tree abstraction problem we consider in this paper.

Information-theoretic frameworks for compression model signals according to their statistical structure. Consequently, we require the introduction of a probability space. To this end, let (Ω,F,P) be a probability space with finite sample space Ω, σ-algebra F and probability measure P. We define the random variables X:Ω→R, Y:Ω→R, and T:Ω→R, where the random variable *X* has probability distribution (mass function) assigned according to p(x)=P({ω∈Ω:X(ω)=x}), with the mass functions for *Y* and *T* defined analogously.

### 3.1. Mutual Information

Given two distributions p(x) and ν(x) over the same set of outcomes, the Kullback-Leibler (KL) divergence between the distributions p(x) and ν(x) is

(1) $$D_{\mathrm{KL}}\left(p\left(x\right),\nu \left(x\right)\right)≜\sum_{x}p\left(x\right)\log\frac{p(x)}{\nu (x)}.$$

The KL-divergence is non-negative and equals zero if and only if p(x)=q(x) for all outcomes *x* [17]. The mutual information between two random variables *X* and *T* is defined in terms of the KL-divergence as

(2) $$I\left(T;X\right)≜D_{\mathrm{KL}}\left(p\left(t,x\right),p\left(t\right)p\left(x\right)\right)=\sum_{t,x}p\left(t,x\right)\log\frac{p(t,x)}{p(t)p(x)}.$$

The mutual information is symmetric (i.e., I(T;X)=I(X;T)), non-negative, and equals zero if and only if p(t,x)=p(t)p(x). The mutual information plays an important role in signal compression theory where it represents the code rate, or average number of bits per source symbol. Consequently, if *T* is a compressed representation (or reproduction) of *X*, then lower values of I(T;X) correspond to greater degrees of achieved compression. In the more general setting, the mutual information is a measure of the degree of statistical correlation between the random variables *X* and *T*, where I(T;X)=0 if and only if *X* and *T* are independent. The mutual information also satisfies

(3) I(T;X)=H(X)−H(X|T)=H(T)−H(T|X),

where H(X) is the Shannon entropy (the Shannon entropy of the random variable *X* is $H\left(X\right)=-\sum_{x}p\left(x\right)\log p\left(x\right)$) of the random variable *X* and H(T|X) is the conditional entropy, measuring the average uncertainty in *T* when given knowledge of *X*. Lastly, the Jensen-Shannon divergence between a collection of probability distributions $\left\{p_{1}\left(x\right),\ldots ,p_{n}\left(x\right)\right\}$ with weights $\Pi \in R_{+}^{n}$ is given by

(4) $${\mathrm{JS}}_{\Pi }\left(p_{1}\left(x\right),\ldots ,p_{n}\left(x\right)\right)=\sum_{i=1}^{n}{\left[\Pi \right]}_{i}D_{\mathrm{KL}}\left(p_{i}\left(x\right),\bar{p}\left(x\right)\right),$$

where $0\leq {\left[\Pi \right]}_{i}\leq 1$ for all i∈{1,…,n}, $\sum_{i=1}^{n}{\left[\Pi \right]}_{i}=1$ and $\bar{p}\left(x\right)=\sum_{i=1}^{n}{\left[\Pi \right]}_{i}p_{i}\left(x\right)$ [22,39,40].

### 3.2. Trees and Trees as Encoders

Our goal in this paper is to leverage information-theoretic signal compression principles in order to generate abstractions for autonomous systems in the form of multi-resolution tree structures. However, existing frameworks for signal encoding, such as rate-distortion theory [17] or the information bottleneck (IB) method [18], do not impose any structural constraints on the resulting encoder in order to guarantee that the solution corresponds to a tree representation. The added constraint poses a significant challenge, as existing methods do not consider such limitations on the set of feasible encoders. To tackle this problem, we will elucidate how trees can be viewed as encoders with a specific structure.

**Figure 1:**
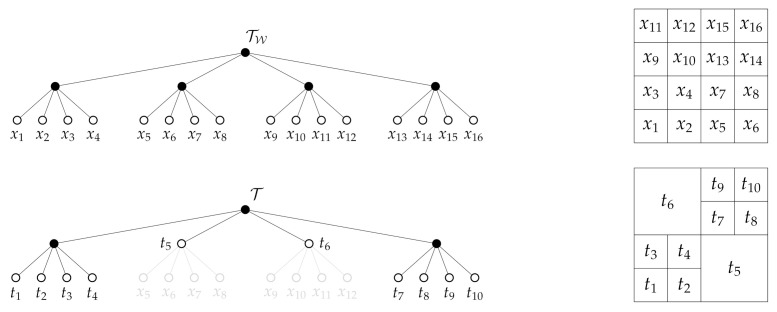
(**top**) The tree $T_{W}$ together with 4×4 grid world representation. (**bottom**) Multi-resolution abstraction of the world W in the form of a quadtree. Notice that the tree $T\in T^{Q}$ is formed by aggregating finest resolution cells that are leafs of $T_{W}$ to their parent nodes, which are leafs of T. Aggregated nodes are shown in grey shading. In both figures, black filled nodes are those nodes that are part of the set $N_{\mathrm{int}}\left(\cdot \right)$, whereas nodes with no fill comprise the set $N_{\mathrm{leaf}}\left(\cdot \right)$.

We assume that the environment $W\subset R^{d}$ is a *d*-dimensional grid-world and that there is an integer ℓ>0 such that the environment is contained within a hypercube of side length $2^{\ell }$. A hierarchical, multi-resolution depiction of W can be represented as a tree (a tree is a connected acyclic graph [41]) T=(N(T),E(T)) where N(T) is a collection of nodes and E(T) is a collection of edges that describe the nodal interconnections [41]. We will henceforth limit the discussion to the case when the tree structure is that of a quadtree, however it should be noted that the theory developed in this paper applies straightforwardly to general tree structures. Given an environment W, we will take $T^{Q}$ to denote the set of all feasible quadtree representations of W, and let $T_{W}\in T^{Q}$ be the tree whose leafs define the finest resolution depiction of W. An example is shown in Figure 1. In the sequel, we follow the notation and definitions of [36,37]. To this end, we let $N_{k}\left(T_{q}\right)$ denote all the nodes of the tree $T_{q}\in T^{Q}$ at depth k∈{0,…,ℓ}, and, for any $t\in N(T_{W})$, C(t) will denote the set of children of *t*. The set of leafs of $T_{q}\in T^{Q}$ is given by $N_{\mathrm{leaf}}\left(T_{q}\right)$ and the interior node set is $N_{\mathrm{int}}\left(T_{q}\right)=N\left(T_{q}\right)\setminus N_{\mathrm{leaf}}\left(T_{q}\right)$.

Given a formal definition of a tree, we are now ready to discuss the connection between hierarchical trees and signal encoders. To this end, it was noted in [36,37] that hierarchical tree abstractions of W can be viewed as deterministic encoders having a specific structure. To this end, notice from Figure 1 that by changing the tree $T\in T^{Q}$ we alter the multi-resolution representation of the environment W. Moreover, any tree $T\in T^{Q}$ can be created by aggregating finest resolution cells to some parent node in the tree in such a way that the resulting tree is in the space $T^{Q}$.

To make the connection to an information-theoretic framework for compression more precise, we let X:Ω→R be the random variable corresponding to the uncompressed signal. In our setting, the uncompressed signal can be the original map of the environment, and therefore the outcomes of *X* are the finest resolution grid cells of W. For example, in the full-resolution (4×4) environment $T_{W}$ depicted in Figure 1, we have $X:\Omega \to \{x_{1},\ldots ,x_{16}\}$. Notice that each tree $T_{q}\in T^{Q}$ defines a compressed random variable $T_{q}:\Omega \to R$ whose outcomes are the elements of the set $N_{\mathrm{leaf}}\left(T_{q}\right)$. The relationship between *X* and $T_{q}$ can be characterized by a deterministic encoder $p_{q}\left(t|x\right)$ where $p_{q}\left(t|x\right)=1$ if and only if the finest resolution cell $x\in N_{\mathrm{leaf}}\left(T_{W}\right)$ is aggregated to the node $t\in N_{\mathrm{leaf}}\left(T_{q}\right)$ in the tree $T_{q}$. However, it is important to note that not all deterministic encoders correspond to valid tree representations of W, which is a challenge we will discuss in the development of our proposed solution approach.

The observation that a tree can be represented as a deterministic encoder, allows us to express information-theoretic quantities as a function of the tree, described next. Consider, for example, the case when a given joint distribution p(x,y) is provided, describing how the finest resolution cells are correlated with a specified random variable *Y*, which we assume contains task-relevant information. Imagine now that we wish to compress the signal *X* in the form of a hierarchical tree so that the resulting tree is maximally retentive regarding the relevant variable *Y*. The resulting joint distribution $p_{q}\left(t,x,y\right)$ can be computed according to $p_{q}\left(t,x,y\right)=p_{q}\left(t|x\right)p\left(x,y\right)$, which is a function of the tree $T_{q}\in T^{Q}$, where we have employed the fact that $T_{q}$ is conditionally independent of *Y* when given *X*. From this, we note that the distributions $p_{q}\left(t,y\right)$, $p_{q}\left(t,x\right)$, $p_{q}\left(t\right)$, p(x) and p(y) can be obtained via the appropriate marginalization of the joint distribution $p_{q}\left(t,x,y\right)$. Therefore, we can write the mutual information as a function of the tree as

(5) $$I_{X}\left(T_{q}\right)≜I\left(T_{q};X\right)=\sum_{t,x}p_{q}\left(t,x\right)\log\frac{p_{q}\left(t,x\right)}{p_{q}\left(t\right)p\left(x\right)},$$

and

(6) $$I_{Y}\left(T_{q}\right)≜I\left(T_{q};Y\right)=\sum_{t,y}p_{q}\left(t,y\right)\log\frac{p_{q}\left(t,y\right)}{p_{q}\left(t\right)p\left(y\right)},$$

where $I_{X}\left(T_{q}\right)$ quantifies the degree of compression and $I_{Y}\left(T_{q}\right)$ quantifies the amount of relevant information contained in the tree $T_{q}$. In this setting, we note that the distributions p(x) and p(y) do not depend on the tree $T_{q}$, as they can be obtained directly from the input distribution p(x,y). In an analogous manner, one may also define the amount of irrelevant information, represented by a random variable *Z*, contained in the tree $T_{q}\in T^{Q}$ as

(7) $$I_{Z}\left(T_{q}\right)≜I\left(T_{q};Z\right)=\sum_{t,z}p_{q}\left(t,z\right)\log\frac{p_{q}\left(t,z\right)}{p_{q}\left(t\right)p\left(z\right)},$$

where we assume that p(x,y,z) is provided and $p_{q}\left(t,x,y,z\right)=p_{q}\left(t|x\right)p\left(x,y,z\right)$.

The expressions (5)–(7) may be generalized to the case where we have a collection $\left\{Y_{1},\ldots ,Y_{n}\right\}$ of relevant and $\left\{Z_{1},\ldots ,Z_{m}\right\}$ of irrelevant variables, respectively, as follows. Given the joint distribution $p(x,y_{1},\ldots ,y_{n},z_{1},\ldots ,z_{m})$ specifying the correlations between relevant and irrelevant variables, the information of each variable contained in the tree $T_{q}\in T^{Q}$ is given by

(8) $$I_{Y_{i}}\left(T_{q}\right)=I\left(T_{q};Y_{i}\right)=\sum_{t,y_{i}}p_{q}\left(t,y_{i}\right)\log\frac{p_{q}\left(t,y_{i}\right)}{p_{q}\left(t\right)p\left(y_{i}\right)},\;i\in \left\{1,\ldots ,n\right\},$$

and

(9) $$I_{Z_{j}}\left(T_{q}\right)=I\left(T_{q};Z_{j}\right)=\sum_{t,z_{j}}p_{q}\left(t,z_{j}\right)\log\frac{p_{q}\left(t,z_{j}\right)}{p_{q}\left(t\right)p\left(z_{j}\right)},\;j\in \left\{1,\ldots ,m\right\},$$

where $p_{q}\left(t,x,y_{1},\ldots ,y_{n},z_{1},\ldots ,z_{m}\right)=p_{q}\left(t|x\right)p\left(x,y_{1},\ldots ,y_{n},z_{1},\ldots ,z_{m}\right)$. Having related trees to signal encoders and showing how mutual information terms can be written as a function of the tree $T_{q}\in T^{Q}$, we now turn to formally state the problem we consider for the remainder of the paper.

## 4. Problem Formulation

In Section 3, we discussed the relation between trees and signal encoders and showed how the observation that a tree $T_{q}\in T^{Q}$ can be represented as a deterministic encoder $p_{q}\left(t|x\right)$ allows us to quantify the information contained in the tree. With these observations, we can now formally state the problem we consider in this paper.

**Problem** **1.** *Given the environment W, vectors $\beta \in R_{+}^{n}$ and $\gamma \in R_{+}^{m}$, a scalar α≥0 and the joint distribution $p(x,y_{1},\ldots ,y_{n},z_{1},\ldots ,z_{m})$, consider the problem of maximizing*

$$\max_{T_{q}\in T^{Q}}\sum_{i=1}^{n}{\left[\beta \right]}_{i}I_{Y_{i}}\left(T_{q}\right)-\sum_{j=1}^{m}{\left[\gamma \right]}_{j}I_{Z_{j}}\left(T_{q}\right)-\alpha I_{X}\left(T_{q}\right).$$

It should be noted that Problem 1 cannot be solved by applying existing algorithms (e.g., the Blahut-Arimoto algorithm [17,18] or the iterative IB method [18,39]) from signal encoding theory as the set of feasible solutions is discrete, in addition to the presence of the constraint that $p_{q}\left(t|x\right)$ must correspond to a tree $T_{q}\in T^{Q}$. The added constraint poses significant technical challenges, as it is not obvious how this constraint can be represented mathematically so as to render Problem 1 solvable via numerical methods. Moreover, as a result of the discrete nature of $T^{Q}$, it follows that Problem 1 cannot be solved via standard (sub-)gradient approaches from optimization theory, as it belongs to a class of combinatorial optimization problems. Despite these challenges, in the next section we propose a novel and tractable numerical algorithm to find a solution to Problem 1 with theoretical guarantees.

Before proceeding, we provide a few comments regarding the relation of Problem 1 to other areas of research. Namely, Problem 1 is similar to problems considered in the information-theoretic security community [42,43,44] where $\left\{Z_{1},\ldots ,Z_{m}\right\}$ are viewed as private variables whose information content we wish not to disclose to an un-trusted party. In this setting, the value of $I_{Z_{j}}\left(T\right)$ represents the amount of private information disclosed by the tree $T\in T^{Q}$ and the vector of weights $\gamma \in R_{+}^{m}$ encodes the relative cost of private information disclosure, allowing for the privacy variables to be distinguished in their importance of revelation. Alternatively, one may interpret the privacy aspects of Problem 1 via conditional entropy. Using (3) and (7), we can write $I_{Z_{j}}\left(T_{q}\right)=H\left(Z_{j}\right)-H\left(Z_{j}|T_{q}\right)$ and note that $H(Z_{j})$ is constant, given the data $p(x,y_{1},\ldots ,y_{n},z_{1},\ldots ,z_{m})$. Consequently, performing the maximization in Problem 1 encourages solutions $T_{q}\in T^{Q}$ for which $H(Z_{j}|T_{q})$ is as large as possible, amounting to trees that attempt to make $Z_{j}$ and $T_{q}$ independent since $H\left(Z_{j}|T_{q}\right)\leq H\left(Z_{j}\right)$. Then, Fano’s inequality ([17], pp. 37–41) implies that the lower bound of the error probability of any estimator designed to infer $Z_{j}$ from $T_{q}$ increases as a function of $H(Z_{j}|T_{q})$. It follows that when $H(Z_{j}|T_{q})$ large, the probability of error when estimating the value of $Z_{j}$ from $T_{q}$ increases [17,42]. Consequently, information regarding $Z_{j}$ remains protected.

It should be noted that the incorporation of additional irrelevant variables when designing abstractions has been considered in other works. Previous approaches that introduce irrelevance variables when forming abstractions, such as the IBSI method [20], employ the viewpoint that the information provided via $\left\{Z_{1},\ldots ,Z_{m}\right\}$ is general task-irrelevant information, with no motivation from an information-theoretic security standpoint. In the IBSI approach, the incorporation of irrelevant information helps improve the quality of abstractions with respect to the task-relevant variable, as aspects of the task-relevant variable that are correlated with the irrelevant information can be discarded when forming the compressed representations. In summary, we note that, while our formulation given by Problem 1 can be interpreted from an information-theoretic security standpoint, the main motivation for our approach is not one of security. Rather, it is the development of a general information-theoretic framework that allows for both relevant and irrelevant information to be specified and balanced versus compression in the design of multi-resolution tree abstractions for autonomous systems. However, as the discussion above shows, the proposed framework could also be useful in obscuring private information contained in quadtree abstractions.

## 5. Solution Approach

In this section, we discuss an approach to find a solution to Problem 1 and introduce a tractable numerical algorithm that searches for an optimal tree as a function of the weight parameters $\beta \in R_{+}^{n}$, $\gamma \in R_{+}^{m}$, and α≥0. In what follows, it will be convenient to write the objective of Problem 1 in terms of the function $J:T^{Q}\times R_{+}^{n}\times R_{+}^{m}\times R_{+}\to R$, defined by

(10) $$J\left(T_{q};\beta ,\gamma ,\alpha \right)=\sum_{i=i}^{n}{\left[\beta \right]}_{i}I_{Y_{i}}\left(T_{q}\right)-\sum_{j=1}^{m}{\left[\gamma \right]}_{j}I_{Z_{j}}\left(T_{q}\right)-\alpha I_{X}\left(T_{q}\right).$$

Then our problem is one of selecting a tree $T_{\tilde{q}}\in T^{Q}$ such that

(11) $$T_{\tilde{q}}\in \underset{T_{q}\in T^{Q}}{\mathrm{argmax}}J\left(T_{q};\beta ,\gamma ,\alpha \right).$$

The evaluation of the objective (10) for a given tree $T_{q}\in T^{Q}$ may be computationally expensive, as it requires the computation of each joint distribution $p(t,y_{i})$, i∈{1,…,n}, and $p(t,z_{j})$, j∈{1,…,m}, as well as the evaluation of the mutual information terms (8) and (9), each of which requires summation over the sample spaces of $\Omega_{T_{q}}$, $\Omega_{Y_{i}}$ and $\Omega_{Z_{j}}$ for each i∈{1,…,n} and j∈{1,…,m}. Such a computation is especially burdensome if the sample spaces have a large number of elements. Instead, we seek an easier, less computationally costly incremental approach toward evaluating the objective (10) for any $T_{q}\in T^{Q}$.

To this end, we write the objective (10) for any $T_{q}\in T^{Q}$ as

(12) $$J\left(T_{q};\beta ,\gamma ,\alpha \right)=J\left(T_{0};\beta ,\gamma ,\alpha \right)+\sum_{u=0}^{q-1}\left[J\left(T_{u+1};\beta ,\gamma ,\alpha \right)-J\left(T_{u};\beta ,\gamma ,\alpha \right)\right],$$

where $\left\{T_{0},\ldots ,T_{q-1}\right\}\subseteq T^{Q}$ is a collection of trees in the space $T^{Q}$. While the relation (12) is valid for any tree $T_{q}\in T^{Q}$ and any collection $\left\{T_{0},\ldots ,T_{q-1}\right\}\subseteq T^{Q}$, it was noted in [36,37] that when the tree $T_{0}\in T^{Q}$ and the sequence $\left\{T_{0},\ldots ,T_{q-1}\right\}\subseteq T^{Q}$ is selected in a specific way, the objective (12) reduces to a special form. Specifically, if we select $T_{0}\in T^{Q}$ as the tree consisting of a single node where all finest resolution cells are aggregated, and the sequence $\left\{T_{0},\ldots ,T_{q-1}\right\}\subseteq T^{Q}$ is constructed by expanding a leaf node of $T_{u}$ to create $T_{u+1}$ for u∈{0,…,q−1}, then (12) can be expressed in terms of the local changes made in moving from the tree $T_{i}$ to $T_{i+1}$. Formally, when the tree $T_{0}\in T^{Q}$ is selected to be the root tree (the root tree $R_{W}$ is the tree $R_{W}\in T^{Q}$ such that $N_{\mathrm{int}}\left(R_{W}\right)=⌀$), and the sequence ${\left\{T_{u}\right\}}_{u=0}^{q}$ is constructed so that $N\left(T_{u+1}\right)\setminus N\left(T_{u}\right)=C\left(t\right)=\left\{t_{1}^{\prime },\ldots ,t_{4}^{\prime }\right\}$ for some $t\in N_{\mathrm{leaf}}\left(T_{u}\right)$ for all u∈{0,…,q−1}, the objective (12) takes the form

(13) $$J\left(T_{q};\beta ,\gamma ,\alpha \right)=\sum_{s\in N_{\mathrm{int}}\left(T_{q}\right)}\Delta J\left(s;\beta ,\gamma ,\alpha \right),$$

where

(14) $$\Delta J\left(t;\beta ,\gamma ,\alpha \right)=\sum_{i=1}^{n}{\left[\beta \right]}_{i}\Delta I_{Y_{i}}\left(t\right)-\sum_{j=1}^{m}{\left[\gamma \right]}_{j}\Delta I_{Z_{j}}\left(t\right)-\alpha \Delta I_{X}\left(t\right),$$

and $\Delta I_{Y_{i}}\left(t\right)=I_{Y_{i}}\left(T_{u+1}\right)-I_{Y_{i}}\left(T_{u}\right)$, $\Delta I_{Z_{j}}\left(t\right)=I_{Z_{j}}\left(T_{u+1}\right)-I_{Z_{j}}\left(T_{u}\right)$, $\Delta I_{X}\left(t\right)=I_{X}\left(T_{u+1}\right)-I_{X}\left(T_{u}\right)$ are given by

(15) $$\begin{array}{cc} \Delta I_{Y_{i}}\left(t\right) & =p\left(t\right){\mathrm{JS}}_{\Pi }\left(p\left(y_{i}|t_{1}^{\prime }\right),\ldots ,p\left(y_{i}|t_{4}^{\prime }\right)\right),\;i\in \left\{1,\ldots ,n\right\}, \end{array}$$

(16) $$\begin{array}{cc} \Delta I_{Z_{j}}\left(t\right) & =p\left(t\right){\mathrm{JS}}_{\Pi }\left(p\left(z_{j}|t_{1}^{\prime }\right),\ldots ,p\left(z_{j}|t_{4}^{\prime }\right)\right),\;j\in \left\{1,\ldots ,m\right\}, \end{array}$$

(17) $$\begin{array}{cc} \Delta I_{X}\left(t\right) & =p(t)H(\Pi ), \end{array}$$

(18) $$\begin{array}{cc} p(y_{i}|t) & =\sum_{u=1}^{4}{\left[\Pi \right]}_{u}p\left(y_{i}|t_{u}^{\prime }\right), \end{array}$$

(19) $$\begin{array}{cc} p(z_{j}|t) & =\sum_{u=1}^{4}{\left[\Pi \right]}_{u}p\left(z_{j}|t_{u}^{\prime }\right), \end{array}$$

(20) $$\begin{array}{cc} p(t) & =\sum_{u=1}^{4}p\left(t_{u}^{\prime }\right), \end{array}$$

(21) $$\begin{array}{cc} \Pi & =\left[\begin{array}{cccc} \frac{p(t_{1}^{\prime })}{p(t)}, & \frac{p(t_{2}^{\prime })}{p(t)}, & \frac{p(t_{3}^{\prime })}{p(t)}, & \frac{p(t_{4}^{\prime })}{p(t)} \end{array}\right]. \end{array}$$

**Figure 2:**
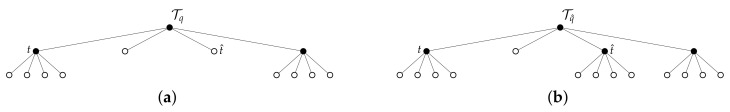
Two trees that differ by only a single leaf node expansion. In moving from tree (**a**) to (**b**), the node $\hat{t}$ is expanded, adding its children as leafs to create the tree shown in (**b**). Interior nodes are shown in black, whereas leaf nodes are white. (**a**) Some tree $T_{q}\in T^{Q}$ for which $\hat{t}\in N_{\mathrm{leaf}}\left(T_{u}\right)$. (**b**) The tree $T_{\hat{q}}\in T^{Q}$ which is created by expanding the node $\hat{t}\in N_{\mathrm{leaf}}\left(T_{u}\right)$.

The relations (15)–(21) are computed via direct calculation in terms of the difference in mutual information between two encoders corresponding to the trees $T_{u+1},T_{u}\in T^{Q}$ that satisfy $N\left(T_{u+1}\right)\setminus N\left(T_{u}\right)=C\left(t\right)$ for some $t\in N_{\mathrm{leaf}}\left(T_{u}\right)$. Observe that the condition $N\left(T_{u+1}\right)\setminus N\left(T_{u}\right)=C\left(t\right)$ for some $t\in N_{\mathrm{leaf}}\left(T_{u}\right)$ implies that the trees $T_{u+1}$ and $T_{u}$ differ only by a single nodal expansion. An example is shown in Figure 2. To show that the term $J(T_{0};\beta ,\gamma ,\alpha )=0$ in (12) when the tree $T_{0}$ is taken to be the root tree, we note from (3) that $0\leq I\left(T_{0};Y_{i}\right)\leq H\left(T_{0}\right)$, $0\leq I\left(T_{0};Z_{j}\right)\leq H\left(T_{0}\right)$ and $0\leq I\left(T_{0};X\right)\leq H\left(T_{0}\right)$. Then, since the root tree has only a single leaf node, it follows that the distribution $p_{0}\left(t\right)$ is deterministic. As a result, $H(T_{0})=0$ and thus $J(T_{0};\beta ,\gamma ,\alpha )=0$.

It is important to note that the incremental relations (15)–(21) depend only on the node $t\in N_{\mathrm{leaf}}\left(T_{u}\right)$ expanded in moving from the tree $T_{u}$ to $T_{u+1}$, and not on any other nodes in the tree. As a result, the evaluation of the incremental changes in information are dependent only on the changes induced by expanding the node $t\in N_{\mathrm{leaf}}\left(T_{u}\right)$, thereby alleviating the need to sum over all the outcomes of the random variable $T_{u}$ as otherwise required in order to evaluate the mutual information. Furthermore, the observation that the objective and information terms can be decomposed into an incremental form according to (13)–(21) allows for tractable algorithms to be designed in order to obtain a solution to Problem 1. Lastly, it is important to note that there is no loss of generality in using the expression (13). To see why this is the case, we present the following definition.

**Definition** **2** ([41]). *A tree G=(N(G),E(G)) is a*subtree*of the tree $H=\left(N(H),E(H)\right)$, denoted by G⊆H, if N(G)⊆N(H) and E(G)⊆E(H).*

Note that the root tree is a subtree of every tree in the space $T^{Q}$. As a result, one can always express the cost (10) as (13), since each tree $T_{q}\in T^{Q}$ can be obtained by starting at the root tree $T_{0}\in T^{Q}$ and creating a sequence ${\left\{T_{u}\right\}}_{u=0}^{q}$ such that $N\left(T_{u+1}\right)\setminus N\left(T_{u}\right)=C\left(t\right)$ for some $t\in N_{\mathrm{leaf}}\left(T_{u}\right)$ and all u∈{0,…,q−1}. Next, we leverage the structure of our problem to design a tractable algorithm in order to find the solution to Problem 1.

### 5.1. The Generalized Tree Search Algorithm (G-Tree Search)

In this section, we show how the structural properties of Problem 1 discussed in the previous section can be exploited in order to yield a tractable algorithm to find a multi-resolution tree that is a solution to (11). Specifically, among all trees $T\in T^{Q}$, we seek those trees that ensure no improvement of (10) is possible, as these trees provide the best trade-off between relevant information retention, irrelevant information removal, and compression. The following definition establishes the notion of optimality we employ throughout this paper.

**Definition** **3.** *A tree $T\in T^{Q}$ is*optimal with respect to *Jif $J\left(\tilde{T};\beta ,\gamma ,\alpha \right)\leq J\left(T;\beta ,\gamma ,\alpha \right)$ for all trees $\tilde{T}\in T^{Q}$.*

To differentiate between candidate solutions, we specify additional properties considered favorable for an optimal multi-resolution tree. One such property is that the tree be minimal, which is defined as follows.

**Definition** **4.** *A tree $T\in T^{Q}$ is*minimal with respect to *Jif $J\left(\tilde{T};\beta ,\gamma ,\alpha \right)<J\left(T;\beta ,\gamma ,\alpha \right)$ for all trees $\tilde{T}\in T^{Q}$ such that $\tilde{T}\subset T$.*

A tree that is both optimal and minimal will be called an *optimal minimal tree*. Importantly, an optimal minimal tree is guaranteed to not contain any redundant nodal expansions. In other words, removing any portion of an optimal minimal tree is guaranteed to result in a pruned tree that is strictly worse with respect to the objective function. In contrast, if an optimal tree is not minimal, then some portion(s) of the tree can be pruned with no loss in the objective value, indicating that the non-minimal tree contains redundant nodal expansions. Thus, of all optimal trees, the minimal solution is preferred as it contains the fewest number of leaf nodes among solution candidates and also requires the least amount of resources to store in memory. Our goal is then to design an algorithm that returns, as a function of $\beta \in R_{+}^{n}$, $\gamma \in R_{+}^{m}$ and α≥0, an optimal minimal tree.

In theory, one may take a number of approaches to find a solution (not necessarily optimal) to Problem 1. One approach is the brute-force method of generating each tree in the space $T^{Q}$ and picking one that satisfies (11); a process which is akin to grid-search methods in optimization theory. However, such an exhaustive approach does not scale well to large environments. Alternatively, one may notice that the node-wise structure of the cost (14) renders the implementation of a greedy approach straightforward. Specifically, given any tree $T_{u}\in T^{Q}$ one may expand the leaf node $t\in N_{\mathrm{leaf}}\left(T_{u}\right)$ that results in the greatest change in the cost ΔJ(t;β,γ,α). By expanding a node $t\in N_{\mathrm{leaf}}\left(T_{u}\right)$, we remove {t} and add its children C(t) to the leaf set to generate the tree $T_{u+1}$, leaving other nodes unchanged. One may continue this process until a tree is reached for which no further improvement is possible, as quantified by the one-step incremental objective value ΔJ(t;β,γ,α). This myopic steepest-ascent-like approach is not guaranteed to find an optimal solution, however, as the process may fail to identify expansions that are suboptimal with respect to the one-step objective ΔJ(t;β,γ,α), but lead to higher-valued expansions in future iterations. Consequently, we seek to incorporate the value of expansions-to-come when deciding whether or not to expand a leaf node of the current tree.

To this end, we introduce a generalized tree search algorithm we call G-tree search. The G-tree search algorithm works from top-down, starting at the root tree $R_{W}\in T^{Q}$ and utilizing the function $G:N\left(T_{W}\right)\times R_{+}^{n}\times R_{+}^{m}\times R_{+}\to R_{+}$, defined as

(22) $$G\left(t;\beta ,\gamma ,\alpha \right)=\left\{\begin{array}{cc} \max\{\Delta J\left(t;\beta ,\gamma ,\alpha \right)+\sum_{t^{\prime }\in C\left(t\right)}G\left(t^{\prime };\beta ,\gamma ,\alpha \right),0\}, & \;\mathrm{if}\;t\in N_{\mathrm{int}}\left(T_{W}\right),\\ 0, & \;\mathrm{otherwise}, \end{array}\right.$$

in order to decide which nodes to expand. Specifically, given any tree $T_{u}\in T^{Q}$, G-tree search will inspect the G-values, computed according to (22), for each node $t\in N_{\mathrm{leaf}}\left(T_{u}\right)$ and expand a node $t\in N_{\mathrm{leaf}}\left(T_{u}\right)$ for which G(t;β,γ,α)>0. Once a node $t\in N_{\mathrm{leaf}}\left(T_{u}\right)$ is selected for expansion, a new tree $T_{u+1}\in T^{Q}$ is defined by removing the node *t* and adding its children, C(t), to the set of leafs, leaving the other nodes in the tree $T_{u}$ unchanged. In this way, the tree $T_{u+1}$ is related to $T_{u}$ via $N_{\mathrm{leaf}}\left(T_{u+1}\right)=\left(N_{\mathrm{leaf}}\left(T_{u}\right)\setminus \left\{t\right\}\right)\cup C\left(t\right)$. The process then repeats until we find a tree $T_{\tilde{q}}\in T^{Q}$ for which there does not exist $t\in N_{\mathrm{leaf}}\left(T_{\tilde{q}}\right)$ such that G(t;β,γ,α)>0. Note that by designing the algorithm in this way, the constraint $T\in T^{Q}$ is naturally enforced. The G-tree search method is detailed in Algorithm 1. Note that the pseudo-code for a greedy tree search is identical to that of G-tree search in Algorithm 1 with each G(t;β,γ,α) replaced by ΔJ(t,β,α,γ). We will discuss the shortcomings of the greedy approach in more detail in Section 6.1.
**Algorithm 1** The G-tree Search Algorithm.
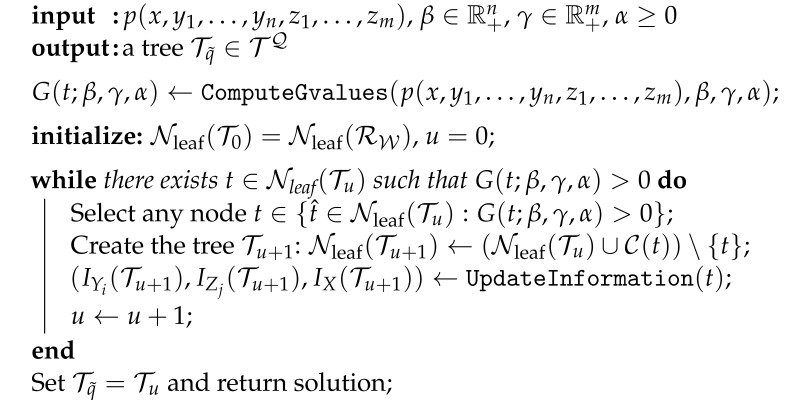


     A few comments are in order regarding the G-tree search method. First, the routine ComputeGvalues(·) populates the G-values, as follows. The routine utilizes the joint distribution $p(x,y_{1},\ldots ,y_{n},z_{1},\ldots ,z_{m})$ in order to compute the values of $\Delta I_{Y_{i}}\left(t\right)$, $\Delta I_{Z_{j}}\left(t\right)$ and $\Delta I_{X}\left(t\right)$ for all i∈{1,…,n}, j∈{1,…,m} and $t\in N_{\mathrm{int}}\left(T_{W}\right)$. Given the weights $\left(\beta ,\gamma ,\alpha \right)\in R_{+}^{n}\times R_{+}^{m}\times R_{+}$ one may compute ΔJ(t;β,γ,α) and apply the rule (22) to obtain the G-values via a recursion that begins at the leafs of $T_{W}$. The pseudo-code for the ComputeGvalues procedure is shown in Algorithm 2. Lastly, the function UpdateInformation(t) updates the information contained in the tree at the current time-step of the solution. It does so by utilizing the values of $\Delta I_{Y_{i}}\left(t\right)$, $\Delta I_{Z_{j}}\left(t\right)$ and $\Delta I_{X}\left(t\right)$ for each i∈{1,…,n} and j∈{1,…,m}, which were computed in the process of evaluating the nodal G-values described above. The information contained in the tree $T_{u+1}$ is then given by $I_{Y_{i}}\left(T_{u+1}\right)=I_{Y_{i}}\left(T_{u}\right)+\Delta I_{Y_{i}}\left(t\right)$, $I_{Z_{j}}\left(T_{u+1}\right)=I_{Z_{j}}\left(T_{u}\right)+\Delta I_{Z_{j}}\left(t\right)$ and $I_{X}\left(T_{u+1}\right)=I_{X}\left(T_{u}\right)+\Delta I_{X}\left(t\right)$ where i∈{1,…,n} and j∈{1,…,m}. Recall that starting the algorithm at the root tree $T_{0}\in T^{Q}$ implies, for all *i* and *j*, we have $I_{Y_{i}}\left(T_{0}\right)=0$, $I_{Z_{j}}\left(T_{0}\right)=0$ and $I_{X}\left(T_{0}\right)=0$.
**Algorithm 2** The ComputeGvalues routine.
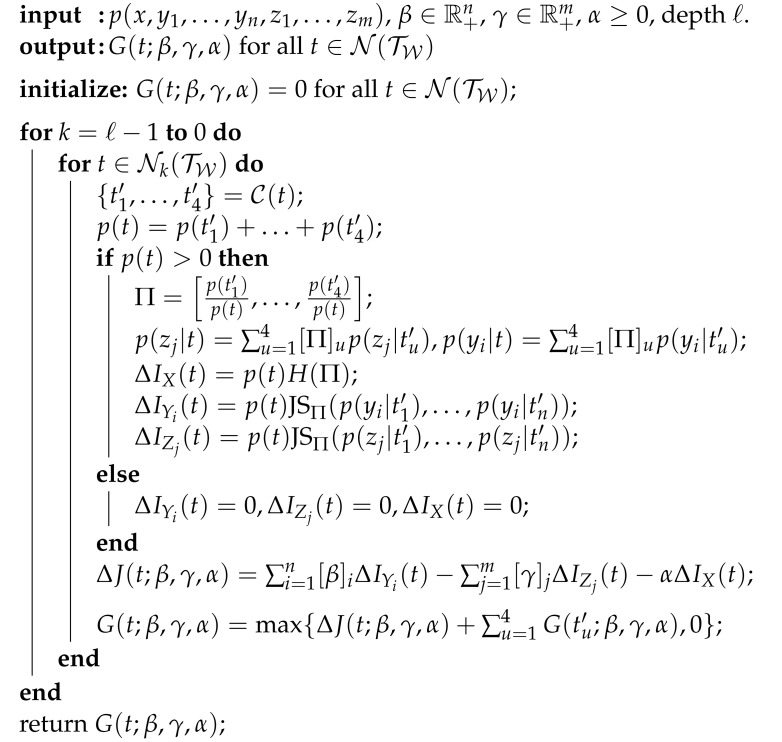


### 5.2. Theoretical Analysis of the G-Tree Search Algorithm

In this section, we discuss the theoretical properties of the G-tree search algorithm introduced in Section 5.1. Our main result is that the G-tree search algorithm returns an optimal minimal tree. In our analysis, we will oftentimes refer to the part of a tree $T_{q}\in T^{Q}$ that is descendant (or rooted) at some node $t\in N(T_{q})$. To make this notion precise, we have the following definition.

**Definition** **5** ([36]). *Let $t\in N(T_{q})$ be a node in the tree $T_{q}\in T^{Q}$.*The subtree of*$T_{q}\in T^{Q}$*rooted at node*t is denoted by $T_{q(t)}$ and has node set*

$$N\left(T_{q(t)}\right)=\left\{t^{\prime }\in N\left(T_{q}\right):t^{\prime }\in \underset{i}{⋃}D_{i}\right\},$$

*where $D_{1}=\left\{t\right\}$, $D_{i+1}=A\left(D_{i}\right)$, and*

$$A\left(D_{i}\right)=\left\{t^{\prime }\in N\left(T_{W}\right):t^{\prime }\in \underset{\hat{t}\in D_{i}}{⋃}C\left(\hat{t}\right)\right\}.$$

**Figure 3:**
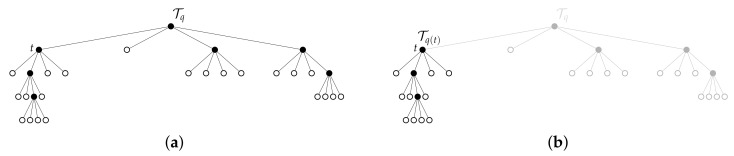
Example of a tree $T_{q}\in T^{Q}$ and the subtree $T_{q(t)}$ rooted at *t*. (**a**) The tree $T_{q}\in T^{Q}$ together with a node $t\in N_{\mathrm{int}}\left(T_{q}\right)$ shown. (**b**) the subtree $T_{q(t)}$ rooted at *t* with original tree $T_{q}$ shown in background.

An example of a subtree is shown in Figure 3. Each time the G-tree search visits and expands a node $t\in N_{\mathrm{int}}\left(T_{W}\right)$, the algorithm can be viewed as determining the part of the subtree rooted at *t* for which a net increase in the objective can be achieved. For example, consider the case when the algorithm is provided with a tree $T_{q}\in T^{Q}$. In order to determine whether or not expanding some $t\in N_{\mathrm{leaf}}\left(T_{q}\right)$ will lead to a tree of greater objective value than $T_{q}$, the algorithm must determine if expanding the node *t* leads to future expansions that result with a tree $T_{\tilde{q}}\in T^{Q}$ for which $J\left(T_{\tilde{q}};\beta ,\gamma ,\alpha \right)>J\left(T_{q};\beta ,\gamma ,\alpha \right)$. Of course, if ΔJ(t;β,γ,α)>0 for some $t\in N_{\mathrm{leaf}}\left(T_{q}\right)$, then it is clear that expanding the node *t* leads to a tree that improves the value of the objective. However, when ΔJ(t;β,γ,α)≤0, the decision of whether or not to expand $t\in N_{\mathrm{leaf}}\left(T_{q}\right)$ is not so clear, as the algorithm must then consider if, by continuing the expansion process along the children of *t*, can result in a tree that improves of the overall objective value. In essence, we are interested in investigating how the G-function in (22) relates to the incremental objective value of a subtree rooted at any $t\in N_{\mathrm{int}}\left(T_{W}\right)$ and to show that, if there exists a subtree rooted at *t* that results in a overall improvement of the objective, then G(t;β,γ,α)>0. To answer this question, we have the following results.

**Lemma** **6.** *Let $t\in N_{\mathrm{int}}\left(T_{W}\right)$, $\beta \in R_{+}^{n},\gamma \in R_{+}^{m}$ and α≥0. Then, $G\left(t;\beta ,\gamma ,\alpha \right)\geq \sum_{s\in N_{\mathrm{int}}\left(T_{q(t)}\right)}\Delta J\left(s;\beta ,\gamma ,\alpha \right)$ for all $T_{q}\in T^{Q}$.*

**Proof.**  The proof is presented in Appendix A. □

**Corollary** **7.** *Let $t\in N_{\mathrm{int}}\left(T_{W}\right)$, $\beta \in R_{+}^{n}$, $\gamma \in R_{+}^{m}$ and α≥0. If there exists a tree $T_{q}\in T^{Q}$ such that $\sum_{s\in N_{\mathrm{int}}\left(T_{q(t)}\right)}\Delta J\left(s;\beta ,\gamma ,\alpha \right)>0$ then G(t;β,γ,α)>0.*

**Proof.**  The result is immediate from Lemma 6. □

As a result of Lemma 6 and Corollary 7, we are guaranteed that, if there is a subtree rooted at *t* for which an increase in the overall objective is possible, then G(t;β,γ,α)>0. Furthermore, we are guaranteed that the value of the G-function (22) is bounded below by the incremental value of the objective contributed by any subtree rooted at *t*. The converse to Lemma 6 and Corollary 7 is also of important; namely if we know G(t;β,γ,α)>0 for some $t\in N_{\mathrm{int}}\left(T_{W}\right)$, then it is of interest in establishing whether or not this implies that there is a subtree rooted at *t* for which a net increase in the objective is possible. This leads us to the following results.

**Lemma** **8.** *Let $t\in N_{\mathrm{int}}\left(T_{W}\right)$, $\beta \in R_{+}^{n}$, $\gamma \in R_{+}^{m}$ and α≥0. If G(t;β,γ,α)>0, then there exists a tree $T_{q}\in T^{Q}$ such that $G\left(t;\beta ,\gamma ,\alpha \right)=\sum_{s\in N_{\mathrm{int}}\left(T_{q(t)}\right)}\Delta J\left(s;\beta ,\gamma ,\alpha \right)$.*

**Proof.**  The proof is presented in Appendix B. □

**Corollary** **9.** *Let $t\in N_{\mathrm{int}}\left(T_{W}\right)$, $\beta \in R_{+}^{n}$, $\gamma \in R_{+}^{m}$ and α≥0. If G(t;β,γ,α)>0, then there exists a tree $T_{q}\in T^{Q}$ such that $\sum_{s\in N_{\mathrm{int}}\left(T_{q(t)}\right)}\Delta J\left(s;\beta ,\gamma ,\alpha \right)>0$.*

**Proof.**  The proof follows from Lemma 8. □

Importantly, Lemma 8 establishes the connection between the G-function and the incremental objective value, as well as the existence of a subtree rooted at $t\in N_{\mathrm{int}}\left(T_{W}\right)$ for which a net positive objective increment is possible, in the case when G(t;β,γ,α)>0. Moreover, Lemma 8 and Corollary 9 together guarantee that if the G-value of a node is strictly positive, then there exists a subtree rooted at the node $t\in N_{\mathrm{int}}\left(T_{W}\right)$ such that expanding *t* (and possibly continuing the expansions process along the children of *t*) will result in a tree that has strictly greater objective value. Also, observe that by combining the results of Corollaries 7 and 9 we obtain the following lemma.

**Lemma** **10.** *Let $t\in N_{\mathrm{int}}\left(T_{W}\right)$, $\beta \in R_{+}^{n}$, $\gamma \in R_{+}^{m}$ and α≥0. Then G(t;β,γ,α)>0 if and only if there exists a tree $T_{q}\in T^{Q}$ such that $\sum_{s\in N_{\mathrm{int}}\left(T_{q(t)}\right)}\Delta J\left(s;\beta ,\gamma ,\alpha \right)>0$.*

**Proof.**  The result is a consequence of Corollaries 7 and 9. □

Lemma 10 is important, as it provides necessary and sufficient conditions linking the existence of a subtree rooted at any node $t\in N_{\mathrm{int}}\left(T_{W}\right)$ to the value of the nodal G-function value. We are now in a position to prove the optimality of the solution returned by G-tree search, as stated by the following theorem.

**Theorem** **11.** *Assume $\beta \in R_{+}^{n}$, $\gamma \in R_{+}^{m}$ and α≥0. Then, the G-tree search algorithm returns an optimal minimal tree with respect to J.*

**Proof.**  The proof is given in Appendix C. □

As a consequence of Theorem 11, we can guarantee that for any set of parameters $\left(\beta ,\gamma ,\alpha \right)\in R_{+}^{n}\times R_{+}^{m}\times R_{+}$, the G-tree search algorithm will return a tree that is the optimal and minimal solution to Problem 1.

### 5.3. Complexity Analysis

While Theorem 11 establishes that the G-tree search algorithm introduced in Section 5.1 returns an optimal minimal tree that satisfies (11), it is also important to characterize the number of operations required to execute the algorithm, in the worst case. To this end, we note that a tree $T_{W}\in T^{Q}$ corresponding to some grid in the *d*-dimensional space with side-length $2^{\ell }$ has $N=\sum_{k=0}^{\ell }2^{dk}$ total nodes, where $N=2^{d\ell }\sum_{k=0}^{\ell }2^{d(k-\ell )}=\left|N_{\mathrm{leaf}}\left(T_{W}\right)\right|\left(\sum_{k=0}^{\ell }2^{d(k-\ell )}\right)\leq \left|N_{\mathrm{leaf}}\left(T_{W}\right)\right|\left(MM2^{d}\;/\;\left(2^{d}-1\right)\right)\leq 2\left|N_{\mathrm{leaf}}\left(T_{W}\right)\right|$ for any integer d>0. As a result, the number of nodes in the tree is on the order of the number of leaf nodes of $T_{W}$. Thus, executing the G-tree search algorithm in Algorithm 1 once the G-function is known, requires order $\left|N_{\mathrm{leaf}}\left(T_{W}\right)\right|$ operations, as the search may visit, in the worst case, every node in the tree. Now, note that for a given number of relevant variables n>0 and irrelevant variables m≥0, the computation of the G-function requires on the order of n+m+2 operations per node in the tree, corresponding to the calculation of $\Delta I_{Y_{i}}\left(t\right),\Delta I_{Z_{j}}\left(t\right)$ and $\Delta I_{X}\left(t\right)$ and G-function values. Thus, visiting each node in the tree requires on the order of $\left(n+m+2\right)|N_{\mathrm{leaf}}\left(T_{W}\right)|$ operations. Consequently, updating the G-values and running the G-tree search requires on the order of $\left(n+m+3\right)|N_{\mathrm{leaf}}\left(T_{W}\right)|$ operations in the worst case for a given setting of the problem.

## 6. The IB and IBSI Principles as Special Cases

In this section, we show how obtaining multi-resolution trees via the information bottleneck (IB) [18] and the information bottleneck with side-information (IBSI) [20] are special cases of Problem 1. Moreover, we establish a relation between these two approaches, showing how a tree solution to the IBSI problem can be obtained from the solution of the IB problem, the latter of which does not consider the removal of irrelevant information from the abstractions. Facilitating a theoretical connection between these two approaches helps us understand the impact of irrelevant information on the resulting tree solutions, and furthermore allows us to incorporate irrelevant information after-the-fact, which is useful in applications when only relevant details are known ahead of time. We begin with a brief overview of the IB and IBSI problems in the context of our problem.

### 6.1. Multi-Resolution Trees via the IB Principle and Drawbacks of Myopic Tree Search

Recall from Section 3 that the IB problem furnishes an approach to design compressed representations of the original signal that are maximally informative regarding task-relevant information. The IB method was first introduced in [18] and considers the optimization problem

(23) $$\min_{p(t|x)}I\left(T;X\right)-\beta I\left(T;Y\right),$$

where the minimization is over all conditional distributions p(t|x), and β≥0 trades the importance of compression and relevant information retention. It is important to note that the original formulation of the IB problem does not impose any constraints on the encoder p(t|x) beyond those required to ensure that p(t|x) is a valid probability distribution. However, as discussed in Section 3, multi-resolution trees can be viewed as deterministic encoders that have special structure. Consequently, one may employ the IB principle in order to generate multi-resolution abstractions that compress the environment and retain task-relevant information, which is given by the problem

(24) $$\max_{T\in T^{Q}}I_{Y}\left(T\right)-\frac{1}{\beta }I_{X}\left(T\right),$$

where (24) is obtained by multiplying (23) by the constant −MM1/β for β>0 and restricting the search to the space $T^{Q}$. Recently, the problem (24) was considered by the authors of [36], who introduce an algorithm called Q-tree search that returns, as a function of β>0, an optimal solution to (24). Interestingly, the Q-tree search algorithm emerges as a special case of the more general G-tree search method developed in this paper by defining

(25) ΔL(t;β)=ΔJ(t;1,0,MM1/β),

and by taking

(26) Q(t;β)=G(t;1,0,MM1/β).

As a result, we see that the Q-tree search method in [36] is a special case of G-tree search, where there is only a single relevant variable with unit weight, there is no irrelevant information, and α=MM1/β.

The single variable case also provides some intuition into the differences between G-tree search and an approach that relies on a one-step steepest-ascent (greedy) method to find a solution to (24). The pseudo-code for a greedy tree search is the same as that of G-tree search in Algorithm 1 with each G(t;β,γ,α) replaced with ΔJ(t;β,γ,α). Implementing a greedy approach, a visited node is expanded only if the one-step cost ΔJ(t;β,γ,α)>0. In the single variable case, this means from (25) that

(27) $$\Delta L\left(t;\beta \right)=p\left(t\right)\left[{\mathrm{JS}}_{\Pi }\left(p\left(y|t_{1}^{\prime }\right),\ldots ,p\left(y|t_{4}^{\prime }\right)\right)-\frac{1}{\beta }H\left(\Pi \right)\right]>0,$$

or, equivalently (it can be shown that ΔL(t;β)→0 as p(t)→0. See ([36], Proposition 1) for more details), ${\mathrm{JS}}_{\Pi }\left(p\left(y|t_{1}^{\prime }\right),\ldots ,p\left(y|t_{4}^{\prime }\right)\right)-\frac{1}{\beta }H\left(\Pi \right)>0$, where $t_{i}^{\prime }\in C\left(t\right)$ for all i∈{1,…,4}. By changing β>0, we alter the preference between trees that represent highly compressed versions of *X* (low β) and trees that are more informative regarding *Y* (large β). From (27), we see that the critical value of β>0 for the node $t\in N_{\mathrm{int}}\left(T_{W}\right)$, denoted $\beta_{\mathrm{cr}}:N_{\mathrm{int}}\left(T_{W}\right)\to \left[0,\infty \right]$, is given by

(28) $$\beta_{\mathrm{cr}}\left(t\right)=\left\{\begin{array}{cc} \frac{H(\Pi )}{{\mathrm{JS}}_{\Pi }\left(p\left(y|t_{1}^{\prime }\right),\ldots ,p\left(y|t_{4}^{\prime }\right)\right)}, & \;\mathrm{if}\;{\mathrm{JS}}_{\Pi }\left(p\left(y|t_{1}^{\prime }\right),\ldots ,p\left(y|t_{4}^{\prime }\right)\right)>0,\\ \infty , & \;\mathrm{otherwise}, \end{array}\right.$$

where $C\left(t\right)=\left\{t_{1}^{\prime },\ldots ,t_{4}^{\prime }\right\}$. The node-wise critical β-values, $\beta_{\mathrm{cr}}\left(t\right)$, determine at what point the node will be expanded, should it be visited by the greedy search algorithm. Namely, if $\beta \leq \beta_{\mathrm{cr}}\left(t\right)$ then the node $t\in N(T_{W})$ will not be expanded, even if the algorithm were to visit the node during the search. Moreover, we see from (28) that among those nodes for which H(Π) is constant, those that have a greater diversity among the distributions $\left\{p\left(y|t_{1}^{\prime }\right),\ldots ,p\left(y|t_{4}^{\prime }\right)\right\}$ will have lower critical β-values than those nodes with less diversity in the conditional distributions $\left\{p\left(y|t_{1}^{\prime }\right),\ldots ,p\left(y|t_{4}^{\prime }\right)\right\}$. Intuitively, what this means is that nodes that provide more *Y*-information for a fixed amount of *X*-information will be expanded at lower values of β compared with those that provide less *Y*-information (e.g., more homogeneous cells), should these nodes be reached.

**Figure 4:**
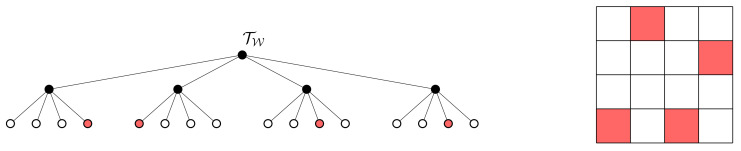
Tree (**left**) and grid (**right**) of a 4×4 example where greedy (myopic) tree search fails. Shading of red scales with p(y|x) (all red shades are equal). Note that, in this environment, parents of leaf nodes contain relevant information, whereas the root node does not, as each of the quadrants is equal in their prediction of *Y* due to environment symmetry.

On the other hand, (28) also shows why a greedy approach may fail to find an optimal solution, even in the single variable setting. To see why this is the case, consider the 4×4 environment in Figure 4. In this example, Y:Ω→{0,1} is the relevant variable, where the grid shading shows the distribution p(y|x). The environment is symmetric in the sense that each of the quadrants contains one cell for which p(y|x)=c for some 0<c≤1. If we assume p(x) to be a uniform distribution, then when *t* is taken to be the root node of $T_{W}$, one will find that ${\mathrm{JS}}_{\Pi }\left(p\left(y|t_{1}^{\prime }\right),\ldots ,p\left(y|t_{4}^{\prime }\right)\right)=0$, H(Π)>0, where $\left\{t_{1}^{\prime },\ldots ,t_{4}^{\prime }\right\}=C\left(t\right)$. Consequently, from (28), we have $\beta_{\mathrm{cr}}\left(t\right)=\infty$. As a result, a greedy implementation will never expand the root node and one will always recover the trivial abstraction, even though there is relevant information in the environment. In contrast, the G-tree search method does not suffer from this drawback, as it incorporates the reward-to-come of future expansions, thereby allowing it to find trees that recover all the relevant information in the environment for finite values of β.

### 6.2. Multi-Resolution Trees via the IB and IBSI Principles

In a similar manner to the IB method considered in Section 6.1, the IBSI approach [20] considers the problem

(29) $$\min_{p(t|x)}I\left(T;X\right)-\beta I\left(T;Y\right)+\gamma I\left(T;Z\right),$$

where the minimization is over all conditional distributions p(t|x), and γ≥0 weights the relative importance of relevant information retention and irrelevant information removal. Constrained to the space of multi-resolution tree abstractions, we obtain the IBSI problem over the space of trees given by

(30) $$\max_{T\in T^{Q}}I_{Y}\left(T\right)-\frac{1}{\beta }\left[\gamma I_{Z}\left(T\right)-I_{X}\left(T\right)\right].$$

The IBSI problem over the space of trees is therefore a special case of Problem 1 where the relevant information has unit weight, the irrelevant information has weight MMγ/β and α=MM1/β for β>0. In (30), the scalar γ≥0 specifies the relative importance of relevant information retention and irrelevant information removal, whereas β>0 balances the importance of compression. We can solve (30) via G-tree search by defining

(31) ΔM(t;β,γ)=ΔJ(t;1,MMγ/β,MM1/β),

and taking the function $S:N\left(T_{W}\right)\times \left(0,\infty \right)\times R_{+}\to R_{+}$ to be given by the rule

(32) S(t;β,γ)=G(t;1,MMγ/β,MM1/β).

We will call the special case of the G-tree search algorithm implemented with the G-function in (32) S-tree search. As a result of (26) and (32), we see that multi-resolution trees via the IB or IBSI principles are obtained by employing the G-tree search. Moreover, the resulting abstractions are guaranteed to be minimal and optimal with respect to their objectives, as specified by Theorem 11.

While (26) and (32) show how trees via the IB and IBSI principles can be obtained as special cases of G-tree search, these relations do not provide us with an understanding of how the introduction of irrelevant information changes the solution of the problem. Thus, to better understand the impact of the presence of irrelevant information on the resulting tree abstractions, we present the following results.

**Lemma** **12.** *Let $t\in N(T_{W})$. Then S(t;β,γ)≤Q(t;β) for all γ≥0 and β>0.*

**Proof.**  The proof is presented in Appendix D. □

**Corollary** **13.** Let γ≥0, β>0 and assume $T_{q_{Q}^{*}},T_{q_{S}^{*}}\in T^{Q}$ are the trees returned by Q-tree search and S-tree search respectively. Then $T_{q_{S}^{*}}\subseteq T_{q_{Q}^{*}}$.

**Proof.**  The proof is presented in Appendix E. □

Corollary 13 essentially states that trees that emerge as a solution to (30) contain no more leaf nodes than those that solve (24), since $T_{q_{S}^{*}}\subseteq T_{q_{Q}^{*}}$. Consequently, for fixed β>0, the presence of irrelevant information works to reduce the number of leaf nodes of the resulting abstraction. This is consistent with the original motivation for the inclusion of irrelevant information discussed in [20]. Namely, the purpose of introducing irrelevant information is so as the improve the quality of the abstractions (or reduce the rate of the code in communication systems) by removing the aspects of the relevant information that are correlated with the irrelevance variable *Z* [20]. In this way, a higher degree of compression can be achieved since we may remove the irrelevant components of the relevant information, a process which is not considered in the IB framework. When applied to multi-resolution tree abstractions, as in our case, this is manifested as a reduction in the number of leaf nodes, as established by Corollary 13.

Having established the influence of irrelevant information on the resulting abstractions, it is also practical to derive an explicit relation between the Q- and S-functions, so that a multi-resolution abstraction that considers irrelevant information can be obtained from the Q-tree search solution. For this result, we define the function $P:N\left(T_{W}\right)\times \left(0,\infty \right)\times \left[0,\infty \right)\to R$ according to the rule

(33) $$\begin{array}{c} P\left(t;\beta ,\gamma \right)=Q\left(t;\beta \right)-\gamma \Delta I_{Z}\left(t\right)-\sum_{t^{\prime }\in B_{t}^{c}}Q\left(t^{\prime };\beta \right)+\sum_{t^{\prime }\in B_{t}}\left[P\left(t^{\prime };\beta ,\gamma \right)-Q\left(t^{\prime };\beta \right)\right], \end{array}$$

when $t\in N_{\mathrm{int}}\left(T_{W}\right)$, where P(t;β,γ)=0 for $t\in N_{\mathrm{leaf}}\left(T_{W}\right)$, and $B_{t}=\left\{t^{\prime }\in C\left(t\right):P\left(t^{\prime };\beta ,\gamma \right)>0\right\}$, $B_{t}^{c}=C\left(t\right)\setminus B_{t}$.

**Lemma** **14.** *Let γ≥0 and β>0. Then P(t;β,γ)≤Q(t;β) for all $t\in N(T_{W})$.*

**Proof.**  The proof is presented in Appendix F. □

We then have the following result.

**Proposition** **15.** *Let γ≥0, β>0. If the function Q(t;β) is known for every $t\in N(T_{W})$ then*

S(t;β,γ)=max{P(t;β,γ),0}.

**Proof.**  The proof is presented in Appendix G. □

Proposition 15 allows us to obtain a multi-resolution tree that incorporates irrelevant information from knowledge of only the Q-function employed by Q-tree search, as well as the values of $\Delta I_{Z}\left(t\right)$ for each $t\in N_{\mathrm{int}}\left(T_{W}\right)$, but does not require $\Delta I_{X}\left(t\right)$ or $\Delta I_{Y}\left(t\right)$ for $t\in N_{\mathrm{int}}\left(T_{W}\right)$. Furthermore, Proposition 15 allows us to incorporate irrelevant information after initially designing a tree that is maximally task-relevant via the IB principle (24). We now turn our attention to demonstrating the utility of the G-tree search method on a non-trivial numerical example.

## 7. Numerical Example and Discussion

**Figure 5:**
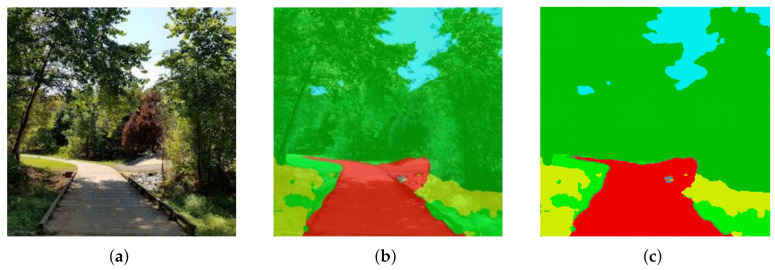
(**a**) Original 256×256 image. (**b**) Segmented 256×256 image with original image in background. (**c**) Segmented 256×256 image passed to G-tree search.

In this section, we demonstrate the utility of our approach on a real-world example. We consider the image shown in Figure 5a, which is of size 256×256. The image in Figure 5a is then segmented, so that each pixel in the original image is classified into one of six distinct categories; the segmented image together with the original image is shown in Figure 5b. Segmented images such as the one shown in Figure 5b arise frequently in autonomous driving scenarios, where it is of interest to remove irrelevant details from the representation so as to focus available resources on only those aspects of the image that are considered important (e.g., the location of the obstacles or the shape of the road). In the segmented image shown in Figure 5c, we see that the task of maintaining relevant information regarding the road corresponds to retaining the red color while the remaining colors, such light green and yellow, are not relevant to the task of identifying the road and should be removed from the representation.

The input data are provided to the G-tree search algorithm as follows. We consider each finest-resolution pixel as an outcome of the uncompressed random variable *X*. Since the agent may not, in general, have the resources (time, computational, etc.) in order to determine the color (or category) information of each pixel with certainty, we model each color in Figure 5c as a random variable. To this end, for each color in Figure 5c we introduce a random variable, where colors that are assumed to be relevant are denoted as $Y_{i}$ and those considered irrelevant as $Z_{j}$. For example, if we would like to generate abstractions where red and blue are relevant (and therefore should be retained) and yellow as irrelevant (and should be removed), we may define $Y_{1}$ to correspond to the category (or color) red and $Y_{2}$ to blue, whereas $Z_{1}$ may represent yellow.

Strictly speaking, knowledge of the distributions $p(y_{i}|x)$, $p(z_{j}|x)$ and p(x) is sufficient to apply our method, as from relations (15)–(21) we see that these distributions allow the determination of $\Delta I_{Y_{i}}\left(t\right)$, $\Delta I_{Z_{j}}\left(t\right)$ and $\Delta I_{X}\left(t\right)$ for all *i*, *j* and *t*. The conditional distributions $p(y_{i}|x)$ and $p(z_{j}|x)$ are obtained from the image segmentation step, where $p(y_{i}|x)$ is the probability that the cell *x* has the color corresponding to $Y_{i}$, with an analogous interpretation for $p(z_{j}|x)$ and $Z_{j}$.In this example, p(x) is assumed to be uniform, although any valid distribution is permissible in our framework (the G-tree search approach can handle any valid distribution p(x) without modification. The use of a non-uniform p(x) will lead to region-specific abstraction, where the G-tree search algorithm refines in regions only where p(x)>0. For more information, the interested reader is referred to [36]). The joint distributions $p(x,y_{i})$ and $p(x,z_{j})$ are then assigned according to $p\left(x,y_{i}\right)=p\left(y_{i}|x\right)p\left(x\right)$ and $p\left(x,z_{j}\right)=p\left(z_{j}|x\right)p\left(x\right)$, respectively. In the more general setting where the input is the joint probability mass function $p(x,y_{1},\ldots ,y_{n},z_{1},\ldots ,z_{m})$, the distributions $p(x,y_{i})$, $p(x,z_{j})$ and p(x) can be obtained via marginalization, and the conditional distributions $p(y_{i}|x)$ and $p(z_{j}|x)$ required to compute (15)–(17) are acquired by applying standard rules for conditional probability.

**Figure 6:**
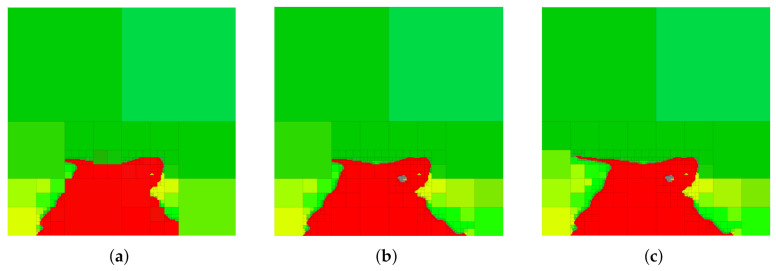
Sample abstractions obtained via the generalized tree search algorithm by defining the color red as relevant and ignoring other colors. In this scenario, the G-tree search method reduces to the IB problem (see Section 6). We assume the (scalar) weighing parameter β=1 and change only α in (10) to generate the abstractions shown. (**a**) α=0.14, $MMI_{Y}\left(T\right)\;/\;I(X;Y)=0.9346$, 0.647% of leaf nodes. (**b**) α=0.05, $MMI_{Y}\left(T\right)\;/\;I(X;Y)=0.9874$, 1.23% of leaf nodes. (**c**) α=0.01, $MMI_{Y}\left(T\right)\;/\;I(X;Y)=1$, 1.42% of leaf nodes.

In order to provide a basis for the discussion that follows, we show in Figure 6 a selection of abstractions obtained by trading relevant information and compression (i.e., the IB problem setting) in the case where red is the relevant variable. A number of observations can be deduced from the abstractions in Figure 6. Firstly, it should be noted that G-tree search finds a tree that retains all the available red information and contains only about 1.42% of the nodes of the finest-resolution space. Next, notice that by changing the parameter α, we change the relative importance of compression and information retention. Consequently, at larger values of α, we obtain abstractions that contain less red information but contain fewer leaf nodes (achieve a greater degree of compression) as compared to the abstractions that arise as α is decreased.

Furthermore, observe from Figure 6 that regions in the image that contain both no red information and are homogeneous in red color remain aggregated even at high values of α. This occurs for two reasons. Firstly, observe from (15) that if a node $t\in N_{\mathrm{int}}\left(T_{W}\right)$ has children $C\left(t\right)=\left\{t_{1}^{\prime },\ldots ,t_{4}^{\prime }\right\}$ for which $p\left(y|t_{1}^{\prime }\right)=\ldots =p\left(y|t_{4}^{\prime }\right)$ for all *y*, then $\Delta I_{Y}\left(t\right)=0$ as ${\mathrm{JS}}_{\Pi }\left(p\left(y|t_{1}^{\prime }\right),\ldots ,p\left(y|t_{4}^{\prime }\right)\right)=0$. Consequently, regions that either contain no red or that are homogeneous in red color contain no relevant information. Intuitively, if a region in the finest resolution is homogeneous in the color red, then no information is lost by aggregating homogeneously-colored finest resolution cells (i.e., given the aggregated cell we can perfectly predict the color of the descendant nodes). Thus, nodes $t\in N_{\mathrm{int}}\left(T_{W}\right)$ for which all descendant nodes are homogeneous in red color provide no additional relevant information, and thus one can see from (22) that G(t;β,γ,α)=0 for these nodes. Notice that the reason regions with no or homogeneous relevant information remain aggregated is due to Theorem 11. To see why, consider the scenario when compression is ignored α=0. In this case, regions that contain no relevant information may be expanded at no cost, but would not contribute to an increase in the objective value as seen by relation (13). However, such expansions would lead to a non-minimal tree to be returned by the G-tree search algorithm, which is precluded by Theorem 11. As a result, G-tree search will return the tree with the least number of leaf-nodes that attains the optimal objective function value. This implies that the tree returned by G-tree search maintains regions with no relevant information aggregated.

**Figure 7:**
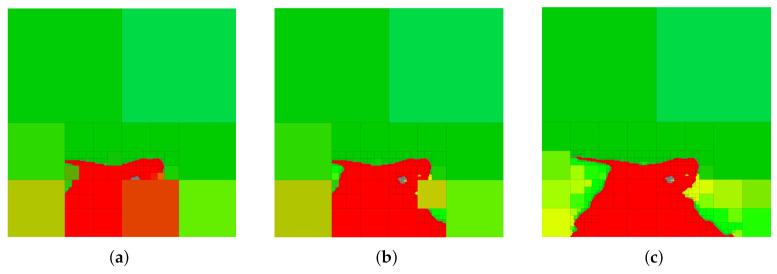
Multi-resolution trees returned by G-tree search in the case when red is relevant and yellow and light green are considered irrelevant for the environment shown in Figure 5. (**a**) Solution for α=0, β=1, ${\left[\gamma \right]}_{1}={\left[\gamma \right]}_{2}=1.2$. (**b**) Solution for α=0, β=1, ${\left[\gamma \right]}_{1}={\left[\gamma \right]}_{2}=0.84$. (**c**) Solution for α=0, β=1, ${\left[\gamma \right]}_{1}={\left[\gamma \right]}_{2}=0$.

Next, we generate multi-resolution tree abstractions by employing G-tree search to not only retain relevant information, but also remove information that is considered irrelevant. To this end, we continue our example of considering red as the relevant variable of interest, now letting light green and yellow be irrelevant variables and represented by $Z_{1}$ and $Z_{2}$, respectively. Example abstractions obtained in this case are shown in Figure 7. Notice that the case shown in Figure 7c corresponds to the standard IB problem with red as relevant and no penalty on compression.

**Figure 8:**
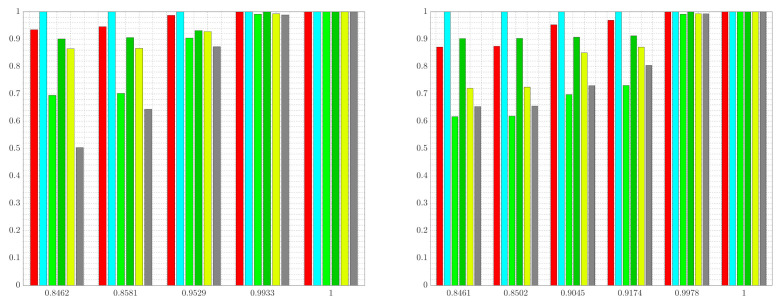
Normalized information retained vs. degree of achieved compression for each color in Figure 5c. Two cases are shown: (i) the standard IB problem with red as relevant (**left**), and (ii) generalized tree search with red as relevant and light green and yellow as irrelevant (**right**). The bar color corresponds with the color in Figure 5c. Data are normalized by the information of each color contained in the tree recovered when executing G-tree search with weights β=1,α=0, and γ=0 (i.e., the tree that retains all the relevant information).

A number of observations can be made from the sample abstractions shown in Figure 7. First, notice that, in comparison with the abstractions shown in Figure 6 which only consider the retention of the color red, the abstractions in Figure 7 aggregate cells along the boundary of red and the irrelevant information (light green and yellow) so as to obscure this information from the abstraction, while being as predictive regarding red (the relevant information) as possible. Moreover, observe that at greater values of the vector γ, regions of yellow and light green are shown in lower resolution as compared with the resolution of these areas at lower values of γ. Notice also that, as the irrelevant information is ignored (${\left[\gamma \right]}_{1}={\left[\gamma \right]}_{2}=0$), we recover an abstraction (Figure 7c) that is equivalent to the tree in Figure 6c returned by the standard IB approach, which does not consider the removal of the irrelevant information content. To better illustrate the differences between the standard IB case shown in Figure 6 and the generalized tree search scenario in Figure 7, we show the normalized information retained by each color for various settings of the weight parameters in Figure 8. The results shown in Figure 8 are obtained by setting β=1, α=0 and by varying the vector $\gamma \in R_{+}^{2}$.

Figure 8 shows the normalized information retained in the solutions returned by G-tree search for two cases: (i) the standard IB problem with red as relevant, and (ii) the generalized tree search with red as relevant and light green as well as yellow as irrelevant. In the standard IB problem, decreasing the value of α≥0 leads to abstractions that are more informative regarding the relevant information, at the cost of obtaining a tree $T\in T^{Q}$ that achieves a lower degree of compression. Consequently, one moves from right to left in Figure 8 (left) as the value of α≥0 is increased. In contrast, in the generalized setting of maintaining red information while removing light green and yellow, increasing the weights of the irrelevant information leads to abstractions that achieve more compression, since the importance of information removal increases with larger values of γ. Thus, keeping all other weights constant, we move from right to left in Figure 8 (right) as $\gamma \in R_{+}^{2}$ is increased.

We also see from Figure 8 that, compared with the IB tree solutions, the trees obtained from the G-tree search approach in case (ii) retain less information regarding light green and yellow. One may also observe from Figure 8 that when the generalized-tree search algorithm is tasked with retaining red information while removing light green and yellow, less red information is retained. This occurs as the importance of information removal necessitates an abstract representation in order to obscure, or remove, the irrelevant details. However, it is only regions that contain both relevant *and* irrelevant information that are of interest to the algorithm in this case, since regions that contain no relevant information are not refined even in the absence of irrelevant information content. In other words, one may view the relevant information as driving refinement, while irrelevant information promoting aggregation. It is therefore regions that contain both irrelevant and relevant information that becomes the focus of G-tree search. We can observe this trend in the abstractions shown in Figure 7. Specifically, notice that regions not containing any relevant information (i.e., regions with no red) are left unchanged and aggregated in Figure 7a–c. In contrast, when comparing the results of Figure 6, where irrelevant information is not taken into account, to those of Figure 7, we see that the areas containing both relevant and irrelevant information are aggregated as the relative importance of information removal is increased. This occurs for the aforementioned reasons, namely, we must sacrifice some relevant information in order to obscure, or remove, the irrelevant details. At the same time, those regions containing red and no irrelevant colors are maintained with relatively high resolution (e.g., the middle of the image where red boarders with darker green), since these regions contain relevant information with no irrelevant details.

**Figure 9:**
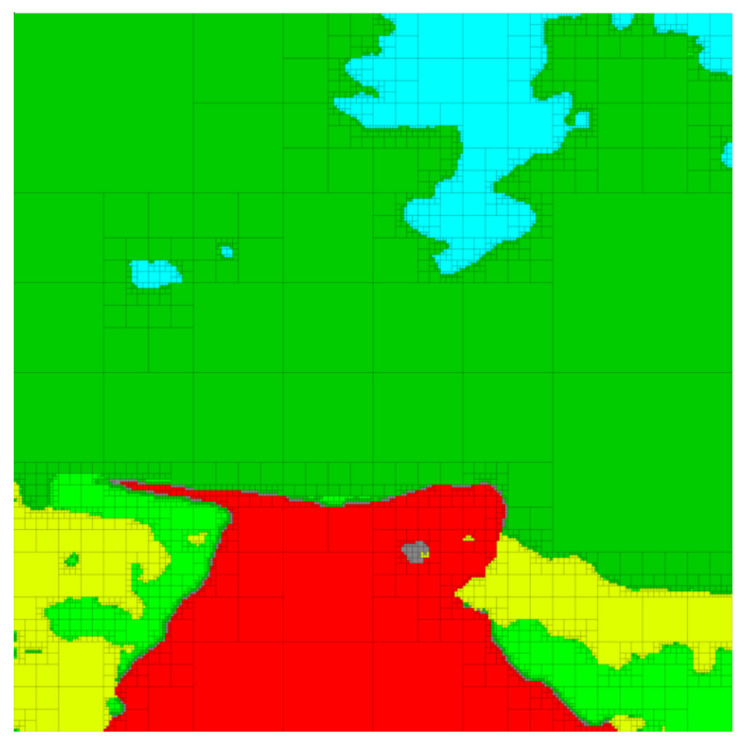
Multi-resolution abstraction of the environment that retains all color information. The image contains only 5% of the nodes compared to the original.

We conclude this section by briefly showcasing the versatility of the G-tree search algorithm to remove redundancies from segmented images. Since the G-tree search algorithm allows any integer number n≥0 of relevant random variables to be defined, it is possible to allow each color in Figure 5c to be a distinct relevant variable. In this case, G-tree search will find trees for which the distinct colors are as distinguishable as possible while balancing the degree of compression achieved by the abstraction. Interestingly, if one were to take ${\left[\beta \right]}_{i}=1$ for all i∈{1,…,6} and α=0, then G-tree search will find a multi-resolution tree that retains all the color information, while removing as much redundancy as possible, as seen in Figure 9. Remarkably, the abstraction in Figure 9 contains only 5% of the nodes of the finest-resolution representation in Figure 5c while retaining *all* the color (semantic) information.

The ability to compress the environment in this way while losing no information regarding the information content of the image represents a drastic savings in the required on-board memory needed to store the depiction of the environment.

## 8. Conclusions

In this paper, we developed a generalized information-theoretic framework for the emergence of multi-resolution abstractions for autonomous agents. To achieve our goal, we formulated the problem of selecting a multi-resolution tree by considering an objective that aims to maximally retain task-relevant information, while simultaneously removes task-irrelevant, or confidential, information and achieves as much compression as possible. Motivated by its use in signal compression theory, we employ the mutual information in order to measure the degree of achieved compression as well as the amount of relevant and irrelevant information retained in the resulting abstract representation. We rigorously investigate the mathematical properties and structure of the problem and discuss the connections between hierarchical tree abstractions and deterministic signal encoders. Moreover, it is shown that the problem we consider has a special structure, whereby the relevant and irrelevant information contained in a hierarchical tree can be expressed in terms of the incremental information contributions of the non-leaf (interior) nodes of the tree. This special, incremental, structure of the problem facilitates the design of the G-tree search algorithm, which searches over the space of multi-resolution abstractions for a solution that maximizes an information-theoretic objective. We detail our proposed algorithm and prove a number of theoretical results, including that the proposed G-tree search algorithm is guaranteed to return an optimal multi-resolution tree. The complexity of the proposed G-tree search algorithm is analyzed and it is shown that multi-resolution tree abstractions via the information bottleneck (IB) method and the information bottleneck problem with side-information (IBSI) are recovered as special cases of our formulation. A non-trivial numerical example is presented to demonstrate the utility of the proposed approach.

## Appendix A

**Proof of Lemma 6.** The proof is given by induction. Consider a node $t\in N_{\ell -1}\left(T_{W}\right)$ and a tree $T_{q}\in T^{Q}$. For the tree $T_{q}$ and the node *t*, there are two options: Either $N_{\mathrm{int}}\left(T_{q(t)}\right)=\left\{t\right\}$ or $N_{\mathrm{int}}\left(T_{q(t)}\right)=⌀$. If $N_{\mathrm{int}}\left(T_{q(t)}\right)=\left\{t\right\}$, then

$$\sum_{s\in N_{\mathrm{int}}\left(T_{q(t)}\right)}\Delta J\left(s;\beta ,\gamma ,\alpha \right)=\Delta J\left(t;\beta ,\gamma ,\alpha \right)\leq \max\left\{\Delta J\left(t;\beta ,\gamma ,\alpha \right),0\right\}=G\left(t;\beta ,\gamma ,\alpha \right).$$

If, instead, $N_{\mathrm{int}}\left(T_{q(t)}\right)=⌀$, then

$$\sum_{s\in N_{\mathrm{int}}\left(T_{q(t)}\right)}\Delta J\left(s;\beta ,\gamma ,\alpha \right)=0\leq \max\left\{\Delta J\left(t;\beta ,\gamma ,\alpha \right),0\right\}=G\left(t;\beta ,\gamma ,\alpha \right).$$

Assume the result holds for all $t^{\prime }\in N_{k}\left(T_{W}\right)$ and some k∈{1,…,ℓ−1}, and consider any node $t\in N_{k-1}\left(T_{W}\right)$ and tree $T_{q}\in T^{Q}$. If $N_{\mathrm{int}}\left(T_{q(t)}\right)\neq ⌀$, then

$$\begin{array}{cc} \sum_{s\in N_{\mathrm{int}}\left(T_{q(t)}\right)}\Delta J\left(s;\beta ,\gamma ,\alpha \right) & =\Delta J\left(t;\beta ,\gamma ,\alpha \right)+\sum_{t^{\prime }\in C\left(t\right)}\sum_{s\in N_{\mathrm{int}}\left(T_{q(t^{\prime })}\right)}\Delta J\left(s;\beta ,\gamma ,\alpha \right),\\ \; & \leq \Delta J\left(t;\beta ,\gamma ,\alpha \right)+\sum_{t^{\prime }\in C\left(t\right)}G\left(t^{\prime };\beta ,\gamma ,\alpha \right), \end{array}$$

where the first step follows from writing the set $N_{\mathrm{int}}\left(T_{q(t)}\right)=\left\{t\right\}\cup \left({⋃}_{t^{\prime }\in C\left(t\right)}N_{\mathrm{int}}\left(T_{q(t^{\prime })}\right)\right)$, and the second step is a result of invoking the induction hypothesis, since $t^{\prime }\in C\left(t\right)\subseteq N_{k}\left(T_{W}\right)$. We then have

$$\begin{array}{cc} \sum_{s\in N_{\mathrm{int}}\left(T_{q(t)}\right)}\Delta J\left(s;\beta ,\gamma ,\alpha \right) & \leq \Delta J\left(t;\beta ,\gamma ,\alpha \right)+\sum_{t^{\prime }\in C\left(t\right)}G\left(t^{\prime };\beta ,\gamma ,\alpha \right),\\ \; & \leq \max\left\{\Delta J\left(t;\beta ,\gamma ,\alpha \right)+\sum_{t^{\prime }\in C\left(t\right)}G\left(t^{\prime };\beta ,\gamma ,\alpha \right),0\right\}=G\left(t;\beta ,\gamma ,\alpha \right). \end{array}$$

If $N_{\mathrm{int}}\left(T_{q(t)}\right)=⌀$, then

$$\begin{array}{cc} \sum_{s\in N_{\mathrm{int}}\left(T_{q(t)}\right)}\Delta J\left(s;\beta ,\gamma ,\alpha \right)=0 & \leq \max\{\Delta J\left(t;\beta ,\gamma ,\alpha \right)+\sum_{t^{\prime }\in C\left(t\right)}G\left(t^{\prime };\beta ,\gamma ,\alpha \right),0\},\\ \; & =G(t;\beta ,\gamma ,\alpha ). \end{array}$$

Thus, $\sum_{s\in N_{\mathrm{int}}\left(T_{q(t)}\right)}\Delta J\left(s;\beta ,\gamma ,\alpha \right)\leq G\left(t;\beta ,\gamma ,\alpha \right)$ for all $T_{q}\in T^{Q}$ and any $t\in N_{\mathrm{int}}\left(T_{W}\right)$. □

## Appendix B

**Proof of Lemma 8.** The proof is given by induction. First, let $t\in N_{\ell -1}\left(T_{W}\right)$ be a node such that G(t;β,γ,α)>0. Next, consider any $T_{q}\in T^{Q}$ such that $N_{\mathrm{int}}\left(T_{q(t)}\right)=\left\{t\right\}$. For any such tree, we have

$$\sum_{s\in N_{\mathrm{int}}\left(T_{q(t)}\right)}\Delta J\left(s;\beta ,\gamma ,\alpha \right)=\Delta J\left(t;\beta ,\gamma ,\alpha \right).$$

Since G(t;β,γ,α)>0, it follows from the definition of G(t;β,γ,α), that

$$G\left(t;\beta ,\gamma ,\alpha \right)=\Delta J\left(t;\beta ,\gamma ,\alpha \right)=\sum_{s\in N_{\mathrm{int}}\left(T_{q(t)}\right)}\Delta J\left(s;\beta ,\gamma ,\alpha \right),$$

and thus, there exits a tree $T_{q}$ such that $G\left(t;\beta ,\gamma ,\alpha \right)=\sum_{s\in N_{\mathrm{int}}\left(T_{q(t)}\right)}\Delta J\left(s;\beta ,\gamma ,\alpha \right)$. Assume now that the result holds for all $t^{\prime }\in N_{k}\left(T_{W}\right)$ and some k∈{1,…,ℓ−1}, and consider any $t\in N_{k-1}\left(T_{W}\right)$ for which G(t;β,γ,α)>0. From the definition of G(t;β,γ,α), we have that

$$\begin{array}{cc} G(t;\beta ,\gamma ,\alpha ) & =\max\{\Delta J\left(t;\beta ,\gamma ,\alpha \right)+\sum_{t^{\prime }\in C\left(t\right)}G\left(t^{\prime };\beta ,\gamma ,\alpha \right),0\},\\ \; & =\Delta J\left(t;\beta ,\gamma ,\alpha \right)+\sum_{t^{\prime }\in C\left(t\right)}G\left(t^{\prime };\beta ,\gamma ,\alpha \right), \end{array}$$

since G(t;β,γ,α)>0. Now, let $U=\{t^{\prime }\in C\left(t\right):G\left(t^{\prime };\beta ,\gamma ,\alpha \right)>0\}$. From the induction hypothesis, it follows that there exists a tree $T_{\hat{q}}\in T^{Q}$ such that $G\left(t^{\prime };\beta ,\gamma ,\alpha \right)=\sum_{s\in N_{\mathrm{int}}\left(T_{\hat{q}\left(t^{\prime }\right)}\right)}\Delta J\left(s;\beta ,\gamma ,\alpha \right)$ for each $t^{\prime }\in U\subseteq C\left(t\right)$. Consider then any tree $T_{q}\in T^{Q}$ such that the subtree rooted at *t* has interior node set

$$N_{\mathrm{int}}\left(T_{q(t)}\right)=\left\{t\right\}\cup \left(\underset{t^{\prime }\in U}{⋃}N_{\mathrm{int}}\left(T_{\hat{q}\left(t^{\prime }\right)}\right)\right).$$

Then,

$$\begin{array}{cc} G(t;\beta ,\gamma ,\alpha ) & =\Delta J\left(t;\beta ,\gamma ,\alpha \right)+\sum_{t^{\prime }\in U}G\left(t^{\prime };\beta ,\gamma ,\alpha \right),\\ \; & =\Delta J\left(t;\beta ,\gamma ,\alpha \right)+\sum_{t^{\prime }\in U}\sum_{s\in N_{\mathrm{int}}\left(T_{\hat{q}\left(t^{\prime }\right)}\right)}\Delta J\left(s;\beta ,\gamma ,\alpha \right)=\sum_{s\in N_{\mathrm{int}}\left(T_{q(t)}\right)}\Delta J\left(s;\beta ,\gamma ,\alpha \right). \end{array}$$

Thus, if G(t;β,γ,α)>0 then there exits a tree $T_{q}\in T^{Q}$ such that $G\left(t;\beta ,\gamma ,\alpha \right)=\sum_{s\in N_{\mathrm{int}}\left(T_{q(t)}\right)}\Delta J\left(s;\beta ,\gamma ,\alpha \right)$. □

## Appendix C

In the following proof, we let $T_{\tilde{q}}\in T^{Q}$ be the tree that is minimal and optimal with respect to *J* and take $T_{G}\in T^{Q}$ denote the tree returned by G-tree search. We will show that $T_{\tilde{q}}=T_{G}$ for all $\beta \in R_{+}^{n},\gamma \in R_{+}^{m}$ and α≥0.

**Proof of Theorem 11.** We begin by showing G(t;β,γ,α)=0 for all $t\in N_{\mathrm{leaf}}\left(T_{\tilde{q}}\right)$. Consider the case when $T_{\tilde{q}}\subset T_{W}$ (notice that if $T_{\tilde{q}}=T_{W}$ then G(t;β,γ,α)=0 for all $t\in N_{\mathrm{leaf}}\left(T_{\tilde{q}}\right)$ by definition). The proof is given by contradiction. Assume that there exists a node $t\in N_{\mathrm{leaf}}\left(T_{\tilde{q}}\right)$ for which G(t;β,γ,α)>0. Then, from Lemma 10, it follows that there exists a tree $T_{\hat{q}}\in T^{Q}$ such that $\sum_{s\in N_{\mathrm{int}}\left(T_{\hat{q}\left(t\right)}\right)}\Delta J\left(s;\beta ,\gamma ,\alpha \right)>0$. Now, consider the tree $T_{q}\in T^{Q}$ with interior node set

$$N_{\mathrm{int}}\left(T_{q}\right)=N_{\mathrm{int}}\left(T_{\tilde{q}}\right)\cup N_{\mathrm{int}}\left(T_{\hat{q}\left(t\right)}\right).$$

It then follows that

$$J\left(T_{q};\beta ,\gamma ,\alpha \right)-J\left(T_{\tilde{q}};\beta ,\gamma ,\alpha \right)=\sum_{s\in N_{\mathrm{int}}\left(T_{\hat{q}\left(t\right)}\right)}\Delta J\left(s;\beta ,\gamma ,\alpha \right)>0,$$

and thus there exists a tree $T_{\hat{q}}\in T^{Q}$ such that $J\left(T_{\hat{q}};\beta ,\gamma ,\alpha \right)>J\left(T_{\tilde{q}};\beta ,\gamma ,\alpha \right)$. However, $T_{\tilde{q}}$ is optimal, leading to a contradiction. Thus, G(t;β,γ,α)=0 for all $t\in N_{\mathrm{leaf}}\left(T_{\tilde{q}}\right)$. As a result, G-tree search will terminate at the leafs of $T_{\tilde{q}}$ should it reach them during the expansion process. However, the algorithm may terminate prior to reaching the leafs of $T_{\tilde{q}}$, and so we conclude $T_{G}\subseteq T_{\tilde{q}}$. Next, we establish that G(t;β,γ,α)>0 for all $t\in N_{\mathrm{int}}\left(T_{\tilde{q}}\right)$. We consider the case when $N_{\mathrm{int}}\left(T_{\tilde{q}}\right)\neq ⌀$ (if $N_{\mathrm{int}}\left(T_{\tilde{q}}\right)=⌀$ then there does not exist $t\in N_{\mathrm{int}}\left(T_{\tilde{q}}\right)$ such that G(t;β,γ,α)=0). The proof is given by contradiction. To this end, assume that there exists a node $t\in N_{\mathrm{int}}\left(T_{\tilde{q}}\right)$ such that G(t;β,γ,α)=0. Since $t\notin N_{\mathrm{leaf}}\left(T_{\tilde{q}}\right)$ and $N_{\mathrm{int}}\left(T_{\tilde{q}}\right)\neq ⌀$, it follows that $N_{\mathrm{int}}\left(T_{\tilde{q}\left(t\right)}\right)\neq ⌀$. Now, consider the tree $T_{q}\in T^{Q}$ with interior node set

$$N_{\mathrm{int}}\left(T_{q}\right)=N_{\mathrm{int}}\left(T_{\tilde{q}}\right)\setminus N_{\mathrm{int}}\left(T_{\tilde{q}\left(t\right)}\right).$$

Then, since $T_{q}\subset T_{\tilde{q}}$ and $T_{\tilde{q}}$ is minimal, it follows that

$$0<J\left(T_{\tilde{q}};\beta ,\gamma ,\alpha \right)-J\left(T_{q};\beta ,\gamma ,\alpha \right)=\sum_{s\in N_{\mathrm{int}}\left(T_{\tilde{q}\left(t\right)}\right)}\Delta J\left(s;\beta ,\gamma ,\alpha \right).$$

Consequently, there exists a tree $T_{\tilde{q}}\in T^{Q}$ such that $\sum_{s\in N_{\mathrm{int}}\left(T_{\tilde{q}\left(t\right)}\right)}\Delta J\left(s;\beta ,\gamma ,\alpha \right)>0$. However, this is a contradiction, since G(t;β,γ,α)=0. Thus, G(t;β,γ,α)>0 for all $t\in N_{\mathrm{int}}\left(T_{\tilde{q}}\right)$. As a result, G-tree search will not terminate prior to reaching the leafs of $T_{\tilde{q}}$, and so $T_{\tilde{q}}\subseteq T_{G}$. We have therefore established that $T_{G}\subseteq T_{\tilde{q}}\subseteq T_{G}$. Thus, $T_{\tilde{q}}=T_{G}$. □

## Appendix D

**Proof of Lemma 12.** The proof is given by induction. Let $t\in N_{\ell -1}\left(T_{W}\right)$. Notice that for node *t*, $C\left(t\right)\subseteq N_{\mathrm{leaf}}\left(T_{W}\right)$. Consequently, $S(t^{\prime };\beta ,\gamma )=0$ and $Q(t^{\prime };\beta )=0$ for all $t^{\prime }\in C\left(t\right)$. From the definitions of *S* and *Q* we then have

S(t;β,γ)=max{ΔM(t;β,γ),0}≤max{ΔL(t;β),0}=Q(t;β),

where the inequality follows from the observation that $\Delta M\left(t;\beta ,\gamma \right)=\Delta L\left(t;\beta \right)-\gamma \Delta I_{Z}\left(t\right)\leq \Delta L\left(t;\beta \right)$ as $\gamma \Delta I_{Z}\left(t\right)\geq 0$. Now assume that the result holds for all $t^{\prime }\in N_{k}\left(T_{W}\right)$, k∈{1,…,ℓ−1} and consider any $t\in N_{k-1}\left(T_{W}\right)$. For node *t* we have, by definition and the induction hypothesis, that

$$\begin{array}{cc} S(t;\beta ,\gamma ) & =\max\{\Delta M\left(t;\beta ,\gamma \right)+\sum_{t^{\prime }\in C\left(t\right)}S\left(t^{\prime };\beta ,\gamma \right),0\},\\ \; & \leq \max\{\Delta M\left(t;\beta ,\gamma \right)+\sum_{t^{\prime }\in C\left(t\right)}Q\left(t^{\prime };\beta \right),0\}, \end{array}$$

since $C\left(t\right)\subseteq N_{k}\left(T_{W}\right)$. Then, since $\Delta M\left(t;\beta ,\gamma \right)=\Delta L\left(t;\beta \right)-\gamma \Delta I_{Z}\left(t\right)\leq \Delta L\left(t;\beta \right)$, we obtain

$$\begin{array}{cc} S(t;\beta ,\gamma ) & \leq \max\{\Delta M\left(t;\beta ,\gamma \right)+\sum_{t^{\prime }\in C\left(t\right)}Q\left(t^{\prime };\beta \right),0\},\\ \; & \leq \max\left\{\Delta L\left(t;\beta \right)+\sum_{t^{\prime }\in C\left(t\right)}Q\left(t^{\prime };\beta \right),0\right\}=Q\left(t;\beta \right), \end{array}$$

thereby establishing that S(t;β,γ)≤Q(t;β). □

## Appendix E

**Proof of Corollary 13.** Notice that Lemma 12 establishes that if, for some $t\in N_{\mathrm{int}}\left(T_{W}\right)$, β>0, and γ≥0, we have S(t;β,γ)>0, then Q(t;β)>0. As a result, any node $t\in N_{\mathrm{int}}\left(T_{W}\right)$ expanded by S-tree search is also expanded by Q-tree search. Thus, $T_{q_{S}^{*}}\subseteq T_{q_{Q}^{*}}$. □

## Appendix F

**Proof of Lemma 14.** The proof is given by induction. First consider any $t\in N_{\ell -1}\left(T_{W}\right)$ and observe that for all such nodes $C\left(t\right)\subseteq N_{\mathrm{leaf}}\left(T_{W}\right)$. Thus, by definition, $Q(t^{\prime };\beta )=0$ and $P(t^{\prime };\beta ,\gamma )=0$ for all $t^{\prime }\in C\left(t\right)$. Therefore,

$$P\left(t;\beta ,\gamma \right)=Q\left(t;\beta \right)-\gamma \Delta I_{Z}\left(t\right)\leq Q\left(t;\beta \right),$$

which follows since $\gamma \Delta I_{Z}\left(t\right)\geq 0$. Assume the result holds for all $t^{\prime }\in N_{k}\left(T_{W}\right)$ and some k∈{1,…,ℓ−1}. Consider now any $t\in N_{k-1}\left(T_{W}\right)$. Then, for the node *t*, we have by definition

$$P\left(t;\beta ,\gamma \right)=Q\left(t;\beta \right)-\gamma \Delta I_{Z}\left(t\right)-\sum_{t^{\prime }\in B_{t}^{c}}Q\left(t^{\prime };\beta \right)+\sum_{t^{\prime }\in B_{t}}\left[P\left(t^{\prime };\beta ,\gamma \right)-Q\left(t^{\prime };\beta \right)\right].$$

Since $B_{t}\subseteq C\left(t\right)\subseteq N_{k}\left(T_{W}\right)$, it follows from the induction hypothesis that

$$P\left(t;\beta ,\gamma \right)\leq Q\left(t;\beta \right)-\gamma \Delta I_{Z}\left(t\right)-\sum_{t^{\prime }\in B_{t}^{c}}Q\left(t^{\prime };\beta \right),$$

since the hypothesis furnishes that $P\left(t^{\prime };\beta ,\gamma \right)-Q\left(t^{\prime };\beta \right)\leq 0$ for all $t^{\prime }\in N_{k}\left(T_{W}\right)$. From the non-negativity of the Q-function and that $\gamma \Delta I_{Z}\left(t\right)\geq 0$, we arrive at

$$P\left(t;\beta ,\gamma \right)\leq Q\left(t;\beta \right)-\gamma \Delta I_{Z}\left(t\right)-\sum_{t^{\prime }\in B_{t}^{c}}Q\left(t^{\prime };\beta \right)\leq Q\left(t;\beta \right),$$

and thus P(t;β,γ)≤Q(t;β) for all $t\in N(T_{W})$. □

## Appendix G

**Proof of Proposition 15.** The proof is given by induction. Consider first any $t\in N_{\ell -1}\left(T_{W}\right)$. For the node *t*, it follows from the definition of S(t;β,γ), that

$$S\left(t;\beta ,\gamma \right)=\max\left\{\Delta M\left(t;\beta ,\gamma \right),0\right\}=\max\left\{\Delta L\left(t;\beta \right)-\gamma \Delta I_{Z}\left(t\right),0\right\},$$

since $S(t^{\prime };\beta ,\gamma )=0$ for all $C\left(t\right)\subseteq N_{\mathrm{leaf}}\left(T_{W}\right)$, and $\Delta M\left(t;\beta ,\gamma \right)=\Delta I_{Y}\left(t\right)-\frac{1}{\beta }\Delta I_{X}\left(t\right)-\gamma \Delta I_{Z}\left(t\right)=\Delta L\left(t;\beta \right)-\gamma \Delta I_{Z}\left(t\right)$. Furthermore, notice that, by definition of P(t;β,γ), we have

$$P\left(t;\beta ,\gamma \right)=Q\left(t;\beta \right)-\gamma \Delta I_{Z}\left(t\right).$$

There are two cases to consider: namely, Q(t;β)>0 and Q(t;β)=0. In the first case (i.e., Q(t;β)>0), we have that Q(t;β)=ΔL(t;β). Consequently,

$$\begin{array}{cc} S\left(t;\beta ,\gamma \right)=\max\{\Delta L\left(t;\beta \right)-\gamma I_{Z}\left(t\right),0\} & =\max\{Q\left(t;\beta \right)-\gamma I_{Z}\left(t\right),0\},\\ \; & =\max\{P(t;\beta ,\gamma ),0\}. \end{array}$$

For the second case, it follows from Lemma 12 that S(t;β,γ)=0. Thus,

S(t;β,γ)=0=max{P(t;β,γ),0},

which holds since, by Lemma 14, P(t;β,γ)≤0 when Q(t;β)=0. Consequently, S(t;β,γ)=max{P(t;β,γ),0} for all $t\in N_{\ell -1}\left(T_{W}\right)$. Assume now that the result holds for all $t^{\prime }\in N_{k}\left(T_{W}\right)$ and some k∈{1,…,ℓ−1}, and consider any $t\in N_{k-1}\left(T_{W}\right)$. For the node *t*, we have, by definition, that

$$S\left(t;\beta ,\gamma \right)=\max\{\Delta M\left(t;\beta ,\gamma \right)+\sum_{t^{\prime }\in C\left(t\right)}S\left(t^{\prime };\beta ,\gamma \right),0\}.$$

Notice that $C\left(t\right)\subseteq N_{k}\left(T_{W}\right)$, and thus, by the induction hypothesis, we have

$$S\left(t;\beta ,\gamma \right)=\max\{\Delta M\left(t;\beta ,\gamma \right)+\sum_{t^{\prime }\in C\left(t\right)}\max\left\{P\left(t^{\prime };\beta ,\gamma \right),0\right\},0\}.$$

Next, define the set $B_{t}=\left\{t^{\prime }\in C\left(t\right):P\left(t^{\prime };\beta ,\gamma \right)>0\right\}$, and write

$$\begin{array}{cc} \; & S\left(t;\beta ,\gamma \right)=\max\{\Delta M\left(t;\beta ,\gamma \right)+\sum_{t^{\prime }\in B_{t}}P\left(t^{\prime };\beta ,\gamma \right),0\}\\ \; & =\max\{\Delta L\left(t;\beta \right)-\gamma \Delta I_{Z}\left(t\right)+\sum_{t^{\prime }\in B_{t}}P\left(t^{\prime };\beta ,\gamma \right),0\},\\ \; & =\max\{\Delta L\left(t;\beta \right)+\sum_{t^{\prime }\in C\left(t\right)}Q\left(t^{\prime };\beta \right)-\sum_{t^{\prime }\in C\left(t\right)}Q\left(t^{\prime };\beta \right)-\gamma \Delta I_{Z}\left(t\right)+\sum_{t^{\prime }\in B_{t}}P\left(t^{\prime };\beta ,\gamma \right),0\}. \end{array}$$

Notice that

$$\begin{array}{cc} \; & \Delta L\left(t;\beta \right)+\sum_{t^{\prime }\in C\left(t\right)}Q\left(t^{\prime };\beta \right)-\sum_{t^{\prime }\in C\left(t\right)}Q\left(t^{\prime };\beta \right)-\gamma \Delta I_{Z}\left(t\right)+\sum_{t^{\prime }\in B_{t}}P\left(t^{\prime };\beta ,\gamma \right)=\\ \; & \Delta L\left(t;\beta \right)+\sum_{t^{\prime }\in C\left(t\right)}Q\left(t^{\prime };\beta \right)-\sum_{t^{\prime }\in S_{t}^{c}}Q\left(t^{\prime };\beta \right)-\gamma \Delta I_{Z}\left(t\right)+\sum_{t^{\prime }\in B_{t}}\left[P\left(t^{\prime };\beta ,\gamma \right)-Q\left(t^{\prime };\beta \right)\right], \end{array}$$

where $S_{t}^{c}=C\left(t\right)\setminus B_{t}$. We now consider the cases when Q(t;β)>0 and Q(t;β)=0. If Q(t;β)>0 then $Q\left(t;\beta \right)=\Delta L\left(t;\beta \right)+\sum_{t^{\prime }\in C\left(t\right)}Q\left(t^{\prime };\beta \right)$, and so we have

$$\begin{array}{cc} \; & \Delta L\left(t;\beta \right)+\sum_{t^{\prime }\in C\left(t\right)}Q\left(t^{\prime };\beta \right)-\sum_{t^{\prime }\in C\left(t\right)}Q\left(t^{\prime };\beta \right)-\gamma \Delta I_{Z}\left(t\right)+\sum_{t^{\prime }\in B_{t}}P\left(t^{\prime };\beta ,\gamma \right)\\ \; & =\Delta L\left(t;\beta \right)+\sum_{t^{\prime }\in C\left(t\right)}Q\left(t^{\prime };\beta \right)-\sum_{t^{\prime }\in S_{t}^{c}}Q\left(t^{\prime };\beta \right)-\gamma \Delta I_{Z}\left(t\right)+\sum_{t^{\prime }\in B_{t}}\left[P\left(t^{\prime };\beta ,\gamma \right)-Q\left(t^{\prime };\beta \right)\right],\\ \; & =Q\left(t;\beta \right)-\sum_{t^{\prime }\in S_{t}^{c}}Q\left(t^{\prime };\beta \right)-\gamma \Delta I_{Z}\left(t\right)+\sum_{t^{\prime }\in B_{t}}\left[P\left(t^{\prime };\beta ,\gamma \right)-Q\left(t^{\prime };\beta \right)\right],\\ \; & =P(t;\beta ,\gamma ), \end{array}$$

where the final equality follows from the definition of P(t;β,γ). Consequently,

$$\begin{array}{cc} \; & S(t;\beta ,\gamma )=\\ \; & =\max\{\Delta L\left(t;\beta \right)+\sum_{t^{\prime }\in C\left(t\right)}Q\left(t^{\prime };\beta \right)-\sum_{t^{\prime }\in C\left(t\right)}Q\left(t^{\prime };\beta \right)-\gamma \Delta I_{Z}\left(t\right)+\sum_{t^{\prime }\in B_{t}}P\left(t^{\prime };\beta ,\gamma \right),0\},\\ \; & =\max\{P(t;\beta ,\gamma ),0\}. \end{array}$$

In the second case, when Q(t;β)=0, we have from Lemma 12 that S(t;β,γ)=0. Furthermore, from Lemma 14 we have P(t;β,γ)≤0. Therefore,

S(t;β,γ)=0=max{P(t;β,γ),0}.

As a result, S(t;β,γ)=max{P(t;β,γ),0} for all $t\in N(T_{W})$. □
